# PARP1 proximity proteomics reveals interaction partners at stressed replication forks

**DOI:** 10.1093/nar/gkac948

**Published:** 2022-11-09

**Authors:** Thorsten Mosler, H Irem Baymaz, Justus F Gräf, Ivan Mikicic, Georges Blattner, Edward Bartlett, Matthias Ostermaier, Rossana Piccinno, Jiwen Yang, Andrea Voigt, Marco Gatti, Stefania Pellegrino, Matthias Altmeyer, Katja Luck, Ivan Ahel, Vassilis Roukos, Petra Beli

**Affiliations:** Institute of Molecular Biology (IMB), Mainz 55128, Germany; Institute of Molecular Biology (IMB), Mainz 55128, Germany; Institute of Molecular Biology (IMB), Mainz 55128, Germany; Institute of Molecular Biology (IMB), Mainz 55128, Germany; Institute of Molecular Biology (IMB), Mainz 55128, Germany; Sir William Dunn School of Pathology, University of Oxford, Oxford, OX1 3RE, UK; Institute of Molecular Biology (IMB), Mainz 55128, Germany; Institute of Molecular Biology (IMB), Mainz 55128, Germany; Institute of Molecular Biology (IMB), Mainz 55128, Germany; Institute of Molecular Biology (IMB), Mainz 55128, Germany; Department of Molecular Mechanisms of Disease, University of Zurich, Zurich CH-8057, Switzerland; Department of Molecular Mechanisms of Disease, University of Zurich, Zurich CH-8057, Switzerland; Department of Molecular Mechanisms of Disease, University of Zurich, Zurich CH-8057, Switzerland; Institute of Molecular Biology (IMB), Mainz 55128, Germany; Sir William Dunn School of Pathology, University of Oxford, Oxford, OX1 3RE, UK; Institute of Molecular Biology (IMB), Mainz 55128, Germany; Institute of Molecular Biology (IMB), Mainz 55128, Germany; Institute of Developmental Biology and Neurobiology (IDN), Johannes Gutenberg-Universität, Mainz, Germany

## Abstract

PARP1 mediates poly-ADP-ribosylation of proteins on chromatin in response to different types of DNA lesions. PARP inhibitors are used for the treatment of *BRCA1/2*-deficient breast, ovarian, and prostate cancer. Loss of DNA replication fork protection is proposed as one mechanism that contributes to the vulnerability of *BRCA1/2*-deficient cells to PARP inhibitors. However, the mechanisms that regulate PARP1 activity at stressed replication forks remain poorly understood. Here, we performed proximity proteomics of PARP1 and isolation of proteins on stressed replication forks to map putative PARP1 regulators. We identified TPX2 as a direct PARP1-binding protein that regulates the auto-ADP-ribosylation activity of PARP1. TPX2 interacts with DNA damage response proteins and promotes homology-directed repair of DNA double-strand breaks. Moreover, TPX2 mRNA levels are increased in *BRCA1/2*-mutated breast and prostate cancers, and high TPX2 expression levels correlate with the sensitivity of cancer cells to PARP-trapping inhibitors. We propose that TPX2 confers a mitosis-independent function in the cellular response to replication stress by interacting with PARP1.

## INTRODUCTION

PARP1 is a member of the PARP family of ADP-ribosyltransferases and an abundant chromatin-associated enzyme in human cells that regulates RNA biogenesis and metabolism as well as chromatin organization and DNA repair (1,2). As part of the immediate response of cells to different types of DNA lesions, PARP1 contributes to the repair of damaged DNA bases, DNA single-strand breaks (SSBs), and double-strand breaks (DSBs). PARP1 promotes faithful SSB repair by initiating base excision repair (BER) or non-homologous end joining (NHEJ) in the case of DNA DSBs (2–4). Binding to damaged DNA activates the catalytic domain of PARP1 and triggers poly-ADP-ribosylation (PARylation) of >700 proteins that function in DNA repair, chromatin remodeling, and RNA metabolism (5–9). In cells, PARP1 forms a complex with Histone PARylation Factor (HPF1) that directs the activity and specificity of PARP1 towards serine residues on its substrates (10–12). Serine-linked ADP-ribosylation was suggested to be the predominant form of protein ADP-ribosylation in response to DNA damage, and can be reversed by the hydrolase ARH3 (13,14). The ADP-ribosylation of Histone H3 at serine 10 and auto-modification of PARP1 are major ADP-ribosylation events after oxidative stress and alkylation damage (11,13,15).

In addition to initiating DNA repair, PARP1 has been reported to play a role in the cellular response to replication stress (16–20). PARP1 binds and gets activated by replication fork-like structures *in vitro* (18). *In vivo*, PARP1 can stabilize stalled replication forks by preventing MRE11-dependent replication fork degradation (16). In addition to its role in replication fork protection, PARP1 acts as a sensor of un-ligated Okazaki fragments, thereby ensuring the proper progression of active replication forks and preventing ssDNA gaps when canonical Okazaki fragment processing is impaired (17,21). Furthermore, PARP1 restrains replication fork speed to avoid excessive exposure of ssDNA gaps behind the replication fork (21–24). As PARP1, the breast cancer susceptibility proteins BRCA1 and BRCA2 also stabilize stalled replication forks. During their canonical roles in HR, BRCA1 promotes DNA end resection by CtIP at DSBs, whereas BRCA2 in association with BRCA1 promotes the replacement of ssDNA-loaded RPA by RAD51 (25–27). At stalled replication forks, BRCA1/2 counteract fork degradation by the MRE11 nuclease (27–29). Furthermore, BRCA1/2 can protect replication forks when forks get susceptible to nucleolytic activity due to PARP inhibition (30).

Synthetic lethality between PARP inhibitors and pathogenic germline mutations in *BRCA1/2* is exploited for the treatment of HR-deficient cancers (31–34). Pathogenic germline mutations in *BRCA1/2* are drivers of breast, ovarian, and prostate cancers (35–38). *BRCA1/2* mutations are estimated to be present in 25% of patients with ovarian cancer and 3% of patients with breast cancer (39–43). The sensitivity of *BRCA*-deficient cells to PARP inhibitors results from a set of traits that leads to the inability to properly respond to DNA damage and replication stress. Defective HR and loss of DNA replication fork protection have been described as mechanisms that make cells with pathogenic *BRCA* mutations susceptible to PARP inhibitors (33,44). Recently, it has been proposed that the accumulation of ssDNA gaps determines the PARP inhibitor sensitivity of cells with BRCA deficiency (21,24). The acquisition of resistance against PARP inhibitors and relapse of patients that responded to treatment remains a major challenge (45,46). Different resistance mechanisms were reported, including the reconstitution of BRCA function, loss of proteins that suppress end resection, restoration of PARylation, prevention of PARP trapping on DNA, suppression of replication gap exposure, and re-establishment of replication fork protection against nuclease-dependent degradation (21,30,44,45,47–56). However, the regulation of PARP1 after replication stress and in the repair of replication-dependent DNA damage remain poorly characterized.

In this study, we set to identify proteins that are in proximity of PARP1 and hence potentially regulate its activity at stressed replication forks. We mapped the PARP1-proximal proteome on native chromatin by fusing full-length PARP1 to an engineered ascorbate peroxidase APEX2 (57). To define which of these proteins play a role with PARP1 at stressed replication forks, we identified proteins recruited to stressed and collapsed replication forks. Proteins involved in DNA repair, RNA biogenesis, and microtubule polymerization were found proximal to PARP1 and stressed replication forks. We demonstrate that TPX2 binds to PARP1 and proteins involved in DNA damage signaling and repair. During mitosis, TPX2 interacts with Aurora kinase A and promotes microtubule polymerization (58–61). We find that TPX2 regulates the ADP-ribosylation activity of PARP1 *in vitro* and *in vivo*. Moreover, *TPX2* mRNA levels are increased in *BRCA1/2*-mutated breast and prostate cancers, and high *TPX2* gene expression levels correlate with the sensitivity of cancer cells to olaparib. Depletion of TPX2 decreases the efficiency of DSB repair through HR and increases genomic instability. We propose that TPX2 confers a mitosis-independent function in stabilizing stressed replication forks by interacting with PARP1 and promoting HR during S-phase.

## MATERIALS AND METHODS

### Cell culture

U2OS and HEK293T cells were obtained from ATCC and cultured in d-MEM medium supplemented with 10% fetal bovine serum, l-glutamine, penicillin, and streptomycin. Cells were routinely tested for mycoplasma infection with a PCR-based method. For SILAC labeling, cells were cultured in media containing either l-arginine and l-lysine, l-arginine [13C6] and l-lysine [2H4] or l-arginine [13C615N4] and l-lysine [13C6-15N2] (Cambridge Isotope Laboratories) as described previously (62). All cells were cultured at 37°C in a humidified incubator containing 5% CO_2_.

### Transfection of siRNA and plasmids

Cells were transfected with siRNAs using Lipofectamine RNAiMAX (Life Technologies) according to the manufacturer's instructions. Plasmid transfections were performed using the PEI transfection reagent (1 mg/ml, Polysciences, Polyethylenimine Max (Mw 4000)) in a 1:3 DNA:PEI ratio.

### Cell lysis

Cells were scraped off the plates in PBS, collected, and pelleted at 250 × g for 5 min. For whole cell extract preparation, cells were lysed in modified RIPA buffer (50 mM Tris pH 7.5, 150 mM NaCl, 1 mM EDTA, 1% NP-40, 0.1% sodium deoxycholate) supplemented with protease inhibitors (Complete protease inhibitor cocktail tablets, Roche Diagnostics), 1 mM sodium orthovanadate, 5 mM β-glycerophosphate, 5 mM sodium fluoride (all from Sigma). Lysates were sonicated and cleared by centrifugation at 16 000 × g for 15 min. For nuclear extract preparation, after collecting cells in PBS, cells were swelled in Buffer A (10 mM KCl, 1.5 mM MgCl_2_, 10 mM HEPES–KOH pH 7.9.) for 10 min on ice and dounce-homogenized (30–35× strokes with pestle B (Kontes)). Nuclei were collected with high-speed centrifugation at 3200 × g for 15 min at 4°C. Nuclei were gently washed with PBS once and then incubated with Buffer C (420 mM NaCl, 20 mM HEPES–KOH pH 7.9, 20% (v/v) glycerol, 2 mM MgCl_2_, 0.2 mM EDTA) on the wheel at 4°C for an h. Nuclear extracts were collected after high-speed centrifugation at 16 000 × g for 50 min. Protein concentrations were estimated using QuickStart Bradford Protein assay (BioRad).

### Proximity biotinylation with APEX2 and Neutravidin pulldown

Cells were transiently transfected with a construct expressing full-length PARP1 tagged on the N-terminus with APEX2 (57). After 48 h, cells were pre-treated with 500 μM biotin phenol (Iris Biochem) for 2 h at 37°C, followed by a 2-min incubation with 1 mM H_2_O_2_ (Sigma-Aldrich) at RT. Cells were washed with quenching solution (10 mM sodium azide, 10 mM sodium ascorbate, 5 mM Trolox (all from Sigma-Aldrich) 3 times and then washed with PBS 3 times. Cell lysis and nuclei isolation was performed as described above. For affinity purification of biotinylated proteins, an equal amount of differentially SILAC labeled extracts, one H_2_O_2_-treated and the other untreated, were combined before the IP and incubated with pre-equilibrated NeutrAvidin agarose beads (Thermo Scientific) for 2 h at 4°C on the wheel. Then the beads were washed once with lysis buffer (modified RIPA or Buffer C depending on the extract used), twice with 8 M Urea (Sigma) and 1% SDS in PBS, and once with 1% SDS in PBS. Bound proteins were eluted in 2 × NuPAGE LDS Sample Buffer (Life Technologies) supplemented with 1 mM DTT and heated at 95°C for 15 min. The eluates, after cooling down to RT, were alkylated by incubating with 5.5 mM chloroacetamide for 30 min in the dark and then loaded onto 4–12% gradient SDS-PAGE gels. Proteins were stained using the Colloidal Blue Staining Kit (Life Technologies) and digested in-gel using trypsin. Peptides were extracted from the gel and desalted on reversed phase C18 StageTips.

### Affinity purification

For GFP affinity purification, differentially SILAC-labeled extracts were incubated with pre-equilibrated GFP Trap beads (Chromotek) for 1 h on the wheel at 4°C followed by five washes with modified RIPA buffer. During the last wash, the beads of the control IP and the specific IP were combined and boiled in 2 × LDS supplemented with 1 mM DTT (70°C for 10 min), followed by alkylation and in-gel digestion. For label-free GFP pull-down, three control and three specific IPs were performed as described, with two additional PBS washes, in the end, followed by on-bead digestion. The stoichiometry of interactions was determined based on label-free pull-down results, using iBAQ intensities as described in Smits *et al.* (63). For endogenous TPX2 affinity purification, pre-equilibrated Dynabeads Protein G (Invitrogen) were coupled to 4 μg of antibody (TPX2 or IgG) for 20 min at RT. The whole cellular lysates were added to the beads and incubated for 2 h at 4°C on the wheel, followed by five washes with modified RIPA buffer. Immunoprecipitated proteins were eluted as described above, run on an SDS-PAGE gel, and transferred to a nitrocellulose membrane for Western blotting.

### SDS-PAGE and western blotting

Proteins were resolved on 4–12% gradient SDS-PAGE gels (NuPAGE® Bis–Tris Precast Gels, Life Technologies) and transferred to nitrocellulose membranes. Membranes were blocked using 10% skimmed milk solution in PBS supplemented with 0.1% Tween-20. Secondary antibodies coupled to horseradish peroxidase (Jackson ImmunoResearch Laboratories) were used for immunodetection. The detection was performed with SuperSignal West Pico Chemiluminescent Substrate (Thermo Scientific).

### Isolation of proteins on nascent DNA (iPOND)

iPOND assays were performed as described (64). Briefly, cells were treated with 10 uM EdU (Jena Bioscience) for 10 min at 37°C. Differently labeled SILAC cells were treated differentially (2 h 2 mM HU treatment, 18 h 2 mM HU treatment, or mock-treated). Then cells were crosslinked with 1% formaldehyde for 20 min at RT, neutralized with 0.125 M glycine, and washed twice with PBS. Cells were scraped off the plates, counted and the same number of cells from each condition were combined and permeabilized with 0.25% Triton X-100 for 30 min at RT. After several washes (1× 0.5% BSA in PBS and 1× PBS), a click reaction was initiated by adding 10 mM sodium ascorbate, 0.1 mM biotin and 2 mM copper sulfate pentahydrate. The samples were rotated on the wheel for 2 h at 4°C, washed 1× 0.5% BSA in PBS and 1× PBS, and lysed with lysis buffer (1% SDS in 50 mM Tris–HCl, pH 8.0) supplemented with protease and phosphatase inhibitors and NEM. After a 15 min incubation on ice, the lysates were sonicated (20 cycles with 30 s on, 90 s off) and clarified by high-speed centrifugation (16 000 × g, 30 min, 4°C). Lysates were diluted 1:1 in PBS and the concentration was estimated using the QuickStart Bradford Protein assay. Lysates were incubated with pre-equilibrated NeutrAvidin agarose beads on the wheel for 1 h at 4°C. Then the beads were washed 2 × with lysis buffer, 1 × with low salt buffer (1% Triton X-100, 20 mM Tris–HCl, pH 8.0, 2 mM EDTA, 150 mM NaCl), 1 × with high salt buffer (1% Triton X-100, 20 mM Tris–HCl, pH 8.0, 2 mM EDTA, 500 mM NaCl), 1 × with LiCl buffer (100 mM Tris–HCl, pH 8.0, 500 mM LiCl, 1% Nonidet P-40) and 2 × with lysis buffer. All washes were performed for 5 min on the wheel at 4°C. Biotinylated proteins were eluted in 100 μl 2 × LDS with 1 mM DTT, boiling at 95°C for 30 min.

### MS analysis

Peptide fractions were analyzed on a quadrupole Orbitrap mass spectrometer (Q Exactive Plus, Thermo Scientific) equipped with a UHPLC system (EASY-nLC 1000, Thermo Scientific) as described (65,66). Peptide samples were loaded onto C18 reversed-phase columns (15 cm length, 75 μm inner diameter, 1.9 μm bead size) and eluted with a linear gradient from 8 to 40% acetonitrile containing 0.1% formic acid for 2 h. The mass spectrometer was operated in data-dependent mode, automatically switching between MS and MS^2^ acquisition. Survey full scan MS spectra (*m*/*z* 300 – 1700) were acquired in the Orbitrap. The 10 most intense ions were sequentially isolated and fragmented by higher-energy C-trap dissociation (HCD) (67). An ion selection threshold of 5000 was used. Peptides with unassigned charge states, as well as with charge state less than +2 were excluded from fragmentation. Fragment spectra were acquired in the Orbitrap mass analyzer.

### Peptide identification

Raw data files were analyzed using MaxQuant (development version 1.5.2.8) (68). Parent ion and MS^2^ spectra were searched against a database containing 92 607 and 96 817 human protein sequences obtained from the UniProtKB released in May 2016 (for iPOND and TPX2 interactome) or February 2020 (for PARP1 proximal proteome), respectively, using Andromeda search engine (69). Spectra were searched with a mass tolerance of 6 ppm in MS mode, 20 ppm in HCD MS^2^ mode, strict trypsin specificity, and allowing up to three miscleavages. Cysteine carbamidomethylation was searched as a fixed modification, whereas protein N-terminal acetylation and methionine oxidation were searched as variable modifications. The dataset was filtered based on posterior error probability (PEP) to arrive at a false discovery rate of below 1% estimated using a target-decoy approach (70).

### Computational analysis

Processed data from MaxQuant were analyzed in RStudio (version 4.1). For the iPOND experiments, SILAC protein ratios were log_2_-transformed and normalized by z-scoring under a normal distribution assumption (*z* = (*x*– mean)/standard deviation). For the PARP1-proximal proteome non-normalized ratios were used. *P*-values for the iPOND experiment and the PARP1-proximal proteome were assessed using LIMMA (71) and corrected for multiple hypothesis testing using the FDR method within LIMMA. For iPOND data, only proteins with an FDR <0.05 and for PARP1-APEX with an FDR <0.01 were considered significantly enriched. Gene Ontology (GO) enrichment analysis was carried out using the R package ViSEAGO (72). *P*-values were calculated with a Fisher's exact test and adjusted for multiple testing using the FDR method. KEGG pathway enrichment was done using EnrichR (73). Protein-protein interactions (PPIs) of 65 overlapping proteins from PARP1-APEX and 18h iPOND were received from the STRING database with a confidence score >0.7. The PPI network was visualized with Cytoscape (version 3.8.2) (74). PFAM domain and Reactome pathway analyses of overlapping proteins were received from STRING. For 558 cell lines, mRNA expression data (CCLE project, (75)) and drug sensitivity data (PRISM Repurposing Primary Screen, (76)) were downloaded from the DepMap portal. Pearson correlation coefficients (PCCs) were calculated for expression values for different genes against relative cell viability in response to treatment with indicated drugs across the CCLE cell line panel. Statistical significance of differences in the distribution of PCCs of different gene sets was assessed using the *t*-test. *z*-scored mRNA expression data (*z*-score of expression of a given gene compared to mean expression of all genes in a given sample) from breast, ovarian and prostate cancer patients with BRCA1 or BRCA2 mutations as well as BRCA wild-type were obtained from the cBioPortal (77,78). The samples were filtered for unique patients. The significance of changes in expression in BRCA1/2-mutated cancers versus BRCA1/2 wild-type cancers was assessed by the t-test using distributions of the *z*-scored expression values of a given gene across the samples of two cancer types (i.e. BRCA1-mutated and wild-type ovarian cancer) and was adjusted for multiple testing using Benjamini-Hochberg correction.

### Extraction of chromatin-associated proteins

Cells were washed with ice-cold PBS and collected using a cell scraper. Cells were lysed in fractionation buffer A (10 mM HEPES pH 7.5, 10 mM KCl, 1.5 mM MgCl2, 0.34 M glucose, 10% glycerol, 1 mM DTT, 0.1% Triton X-100) supplemented with protease and phosphatase inhibitors. Nuclei were pelleted by centrifugation at 1300 × g for 5 min and resuspended in Fractionation buffer B (3 mM EDTA, 0.2 mM EGTA, 1 mM DTT). After incubation, samples were centrifuged at 1700 × g for 5 min and the chromatin pellet was dissolved in the modified RIPA buffer containing 450 mM NaCl. Digestion with benzonase nuclease was used to release chromatin-bound proteins. The lysates were cleared by centrifugation at 16 000 × g for 10 min.

### Cell cycle analysis

Cells were transfected with siRNAs against the proteins of interest or non-targeting siRNAs. 72 h post‐transfection cells were incubated with 10 μM EdU for 1 h before collection and fixed in 4% paraformaldehyde solution. Permeabilization of the cells was performed by incubation in 1% BSA in PBS. Click reaction was performed (Alexa Fluor 647 (Thermo Fisher Scientific), 10 mM (+)‐sodium l‐ascorbate and 2 mM copper (II) sulfate pentahydrate in PBS) for 1 h. Subsequently, cells were incubated for 20 min with 7‐AAD viability staining solution (eBioscience) before analysis by flow cytometry using a BD LSRFortessa SORP.

### Double strand break repair assay

The assay was adapted from Certo *et al.* (79) U2OS cells stably transfected with I-Sce-I cut site were cultured in d-MEM medium supplemented with 10% fetal bovine serum, l-glutamine, penicillin and streptomycin. Cells were transiently transfected with siRNAs against the proteins of interest. 6 h after siRNA transfection, cells were transfected with plasmids expressing the GFP donor and I-Sce-I endonuclease. 72 h later, the cells were trypsinized, washed 2 × with PBS, and measured on the flow cytometer (BD LSRFortessa SORP).

### Single cell electrophoresis

A neutral comet assay was performed according to the manufacturer's protocol (Trevigen). Briefly, cells were embedded in low melting agarose at 37°C on Comet Slides (Trevigen). Overnight cell lysis at 4°C was followed by equilibration in 1 × Neutral Electrophoresis Buffer for 30 min at room temperature. Single-cell electrophoresis was performed at 4°C in 1 × Neutral Electrophoresis buffer for 45 min with a constant 21V. After DNA precipitation with 1 × DNA Precipitation Buffer, Comet Slides were dried with 70% EtOH at room temperature. To completely dry the samples, Comet Slides were transferred to 37°C for 15 min. DNA was stained with SYBR Gold solution for 30 min at room temperature. Images were taken with a Leica AF7000 microscope using a 20 × air objective and a Fluorescein filter. Tail moments of the comets were quantified using the CometScore (TriTek Corp.) software. At least 50 comets were quantified per condition.

### High content microscopy and image analysis

Cells were washed 2 × with PBS, incubated with 0.4% NP-40 for 40 min on ice, fixed with 4% paraformaldehyde in PBS for 15 min, and blocked for 30 min with 5% fetal bovine serum albumin in PBS–Tween (0.1%) containing penicillin and streptomycin. Alternatively, after the PBS washes, cells were incubated with CSK Buffer (10 mM PIPES, 300 mM sucrose, 100 mM NaCl, 3 mM MgCl_2_) containing 0.5% Triton-X, fixed with 4% paraformaldehyde in PBS for 20 min and blocked for 30 min with 3% bovine serum albumin in PBS. Overnight primary antibody incubation at 4°C was followed by 2 × PBS–Tween (0.1%) washes and 1-h incubation with the corresponding secondary antibody coupled to Alexa Fluor 488 or 568 and Hoechst stain. Cells were washed 2 × with PBS–Tween and kept at 4°C in PBS until imaging. Imaging was performed in the Opera Phenix microscope using a 40 × water objective for foci imaging and a 20 × water objective for cell cycle analysis. Image analysis was performed by using Harmony High-Content Imaging and Analysis Software (version 4.4, PerkinElmer). Standard building blocks have allowed to segment nuclei based on the Hoechst signal and cells on the edges of the field were excluded. Mean intensity measurements were performed in maximum projection and spots were detected by using algorithm B (80).

### Site-directed mutagenesis

Q5 site-directed mutagenesis was performed to generate a siRNA-resistant TPX2 cDNA. TPX2 cDNA in a pENTR223 vector was used as a template for exponential amplification. PCR was performed using Q5 polymerase (NEB) and phosphorylated TPX2 K59A/K60A primers. The PCR reaction was followed by DpnI digestion at 37°C for 1 h. Afterward, DNA was purified by PCR clean-up columns (Macherey-Nagel). Purified PCR fragments were treated with Blunt/TA ligase for 15 min at room temperature. Ligated vectors were transformed into chemically competent DH5a bacteria and plated on Spectinomycin LB plates. Successful mutagenesis was confirmed by sequencing.

### Proximity ligation assay (PLA)

Proximity ligation assay was performed according to the manufacturer′s protocol (Duolink^®^, Sigma-Aldrich). Cells were fixed with 4% paraformaldehyde in PBS and permeabilized with 0.25% Triton-X. Samples were blocked with Duolink^®^ Blocking Solution for 1 h at 37°C in a humidity chamber. After removal of the blocking solution, primary antibodies diluted in Duolink^®^ Antibody Diluent were added to the coverslips for 2 h at room temperature in a humidity chamber. Coverslips were washed 2× with Washing Buffer A. PLA Plus and Minus probes were put on in a 1:5 dilution in Duolink^®^ Antibody Diluent for 1 h at 37°C in a humidity chamber. Two washes with Washing Buffer A were followed by ligase treatment in 1× Ligation Buffer for 30 min at 37°C in a humidity chamber. Ligation buffer was tapped off and coverslips were washed twice with Washing Buffer A. Amplification was achieved by adding the Polymerase in 1× Amplification buffer for 100 min at 37°C in a humidity chamber. After washing the samples twice with 1× Washing Buffer B and once with 0.1× Washing Buffer B, coverslips were stained with Hoechst and mounted using Dako mounting medium. Images were taken with a Leica SPE microscope using a 60× oil objective. The number of PLA spots per nucleus was quantified.

### *In vitro* pull downs

GFP-tagged TPX2 mutants were immobilized on pre-equilibrated GFP-Trap beads (Chromotek) in Co-IP buffer (20 mM Tris–HCl pH 7.5, 250 mM NaCl, 0.2% glycerol, 0.2% Triton X-100, and protease inhibitor cocktail) for 1 h at 4°C on a rotating wheel. Equimolar PARP1 was added after one wash with Co-IP buffer and incubated for 2 h at 4°C on a rotating wheel. The beads were washed 4× with Co-IP buffer before being dried with a syringe and boiled in 2× LDS buffer supplemented with 1 mM DTT (95°C for 10 min). Pull downs were subsequently analyzed by Western blot.

### *In vitro* PARylation assay

*In vitro* PARylation assays were performed as described with the inclusion of recombinant TPX2 protein and Histone H3 peptide as noted in the figures (81). Recombinant PARP1, HPF1, and TPX2 concentrations were 1 μM and histone peptides were used at 0.5 μg per reaction. The PARP reaction buffer contained 50 mM Tris–HCl pH 8.0, 100 mM NaCl, 2 mM MgCl_2_, activated DNA and 50 μM NAD+ spiked with 32PNAD+. The modification reaction was incubated at room temperature for 20 min before the addition of 1 μM PARP inhibitor olaparib. Reactions were then analyzed by SDS-PAGE and autoradiography or western blotting.

### List of antibodies used in this study

**Table:** 

**Protein/PTM**	**Product number**	**Manufacturer**	**Dilution (WB/IF)**
GFP	sc-9996	Santa Cruz	1:2000
FLAG	F1804	Sigma	1:2000
TPX2 (D2R5C)	#12245	Cell Signaling	1:1000
PARP1 (46D11)	#9532	Cell Signaling	1:1000
PARP1	sc-8007	Santa Cruz Biotechnology	1:1000
CHD1L (E1I8C)	#13460	Cell Signaling	1:1000
Ku70 (D10A7)	4588S	Cell Signaling	1:1000
PAR	4335-MC-100	Trevigen	1:1000
pan-ADP-Ribose	MABE1016	Merck	1:1000
B-actin	A2228	Sigma	1:10 000
Histone H2A (L88A6)	3636	Cell Signaling	1:1000
p-RPA32 (S4/S8)	A300-245A	Bethyl	1:1000
Streptavidin-HRP	21130	Thermo Scientific	1:10 000
53BP1	MAB3802	Millipore	1:1000
γH2AX	A300-081A-M	Bethyl	1:1000/1:400
HPF1	HPA043467	Sigma Aldrich	1:1000/1:500
RPA70	ab176467	Abcam	–/1:250

### List of siRNA used in this study

**Table:** 

**Gene name**	**Sequence 5′-3′**
TPX2 1	GAAUGGAACUGGAGGGCUU
	AAGCCCUCCAGUUCCAUUC
TPX2 2	AAGGAGAUACUCAAAACAUAG
	CUAUGUUUUGAGUAUCUCCUU
TPX2 3	TGACAACACTTACTACAAA
	UUUGUAGUAAGUGUUGUCA
Control	UGGUUUACAUGUUGUGUGA
	UCACACAACAUGUAAACCA
CtIP	GCUAAAACAGGAACGAAUC
	GAUUCGUUCCUGUUUUAGC
Si control pool	D-001820–10 – Horizon Discovery
TPX2 pool	L-010571–00 – Horizon Discovery
HPF1 pool	L-020849–02 – Horizon Discovery

### List of oligos used in this study

**Table:** 

**Construct**	**Sequence 5′-3′**
TPX2 K59A/K60A	ggtggcctaTTTCAGGGCAAAACTCCTTTG
	tgtaccgtttTTCCCCAGTAACTTATTCTC
TPX2_254only	TACCCAACTTTCTTGTACA
	GACCTGGCTCACTGAT

## RESULTS

### Charting the PARP1-proximal proteome

**Figure 1.**
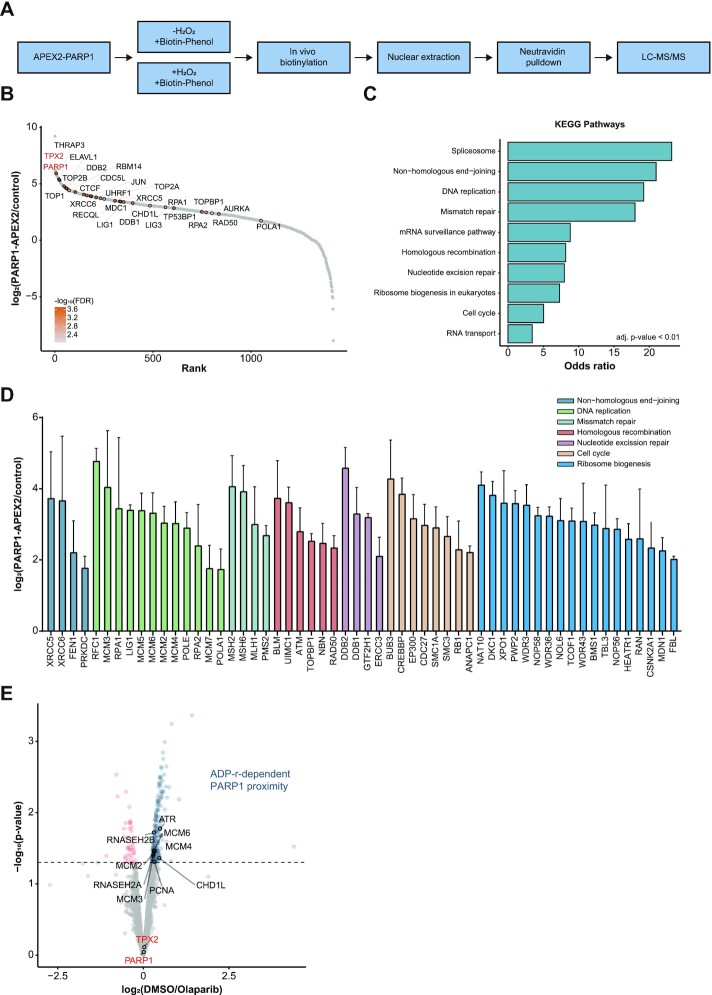
Charting the PARP1-proximal proteome. (**A**) PARP1 fused via its N-terminus to APEX2 was expressed in SILAC-labelled U2OS cells. Biotinylation of proximal proteins was induced by adding 500 μM biotin phenol for 2 h and 1 mM H_2_O_2_ for 2 min. Nuclear biotinylated proteins were enriched using Neutravidin and analyzed by LC–MS/MS. Un-induced condition without H_2_O_2_ served as a control. (**B**) Ranking plot of PARP1 proximal proteins based on the mean ratio of *n* = 4 biologically independent experiments containing a SILAC label switch. Proteins of interest are highlighted and their corresponding –log_10_(FDR) is represented as a color gradient from grey to orange. (**C**) Bar plot representing the top 10 enriched KEGG pathways of PARP1 proximal proteins with an FDR < 1%. Pathways are ranked based on their odds ratio from EnrichR (73). All represented terms have an adjusted *P*-value < 0.01 calculated using Fisher′s exact test with Bonferroni correction. (**D**) Enrichment of PARP1-proximal proteins contributing to the KEGG pathways in (C). Data are represented as mean ± standard deviation. Colors indicate the depicted KEGG pathways. Proteins contributing to RNA metabolism KEGG pathways are displayed in Supplementary Figure S1c. (**E**) Volcano plot displaying the mean ratio and the corresponding –log_10_(*P*-value) of *n* = 3 biologically independent experiments of PARP1 APEX2 experiments after olaparib or DMSO treatment in HEK293T cells. Proteins that show decreased proximity to PARP1 after olaparib treatment are depicted in blue, while proteins that show increased proximity after olaparib treatment are shown in red.

**Figure 2.**
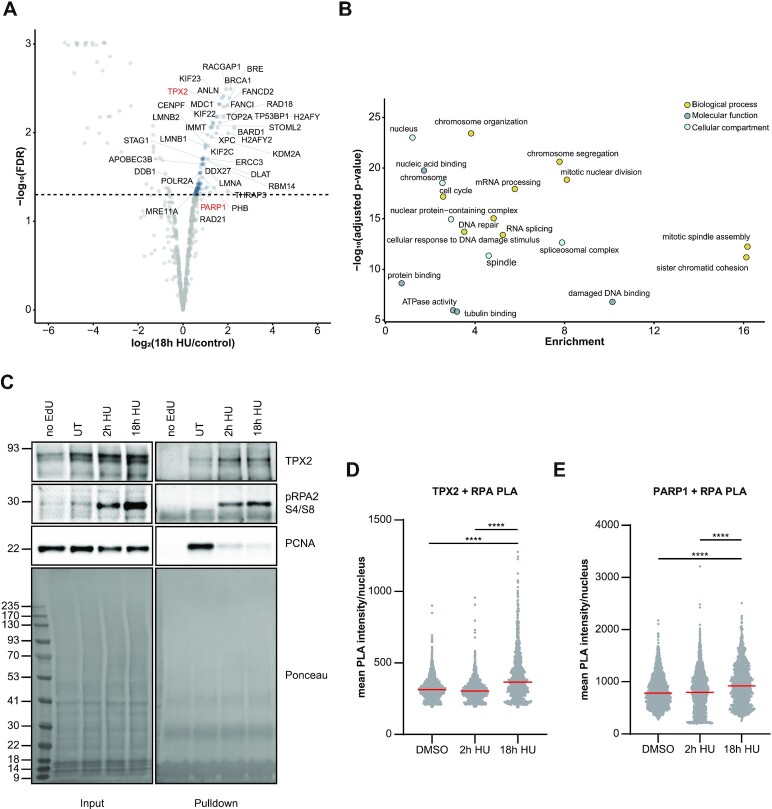
Proteins recruited to persistently stalled replication forks. (**A**) Volcano plot displaying the results from *n* = 2 biologically independent SILAC iPOND experiments after 18 h hydroxyurea (HU) treatment against untreated control. FDR was calculated using limma. Selected proteins with an FDR < 5% are highlighted. (**B**) GO term analysis (Molecular Function, Biological Process and Cellular Compartment) of proteins enriched at persistently stalled replication forks (18 h HU) with an FDR < 5%. *P*-values were calculated by Fisher′s exact test and corrected for multiple comparisons using Benjamini–Hochberg correction. (**C**) Western blot validation of indicated proteins enriched by iPOND after 2 or 18 h hydroxyurea (HU) treatment. (**D**) Representative box plot of a proximity ligation assay (PLA) of *n* = 2 biologically independent experiments between TPX2 and RPA2 after treatment with DMSO or hydroxyurea (HU) for 2 or 18 h. Individual measurements of the mean PLA intensity per nucleus are plotted and the median indicated in red. *P*-values (****P-value < 0.0001) were derived from using one-way-ANOVA with Tukey correction for multiple comparisons. (**E**) Representative box plot of a proximity ligation assay (PLA) of *n* = 2 biologically independent experiments between PARP1 and RPA2 after treatment with DMSO or hydroxyurea (HU) for 2 or 18 h. Individual measurements of the mean PLA intensity per nucleus are plotted and the median indicated in red. *P*-values (****P-value < 0.0001) were derived from using one-way-ANOVA with Tukey correction for multiple comparisons.

To identify the PARP1-proximal proteome in human osteosarcoma (U2OS) cells, we fused full-length PARP1 to an engineered ascorbate peroxidase APEX2. Proximal proteins were labeled in their native chromatin environment with biotin and biotinylated proteins were enriched and subsequently identified by quantitative mass spectrometry (MS) (57). Stable isotope labeling with amino acids in cell culture (SILAC) was used to determine proteins that were specifically labeled upon a short H_2_O_2_ pulse (1 mM, 2 min), which is required to activate APEX2, compared to endogenously biotinylated proteins (Figure 1A, Supplementary Table S1). We enriched nuclear and chromatin-associated proteins in four biologically independent experiments to focus on PARP1 regulators in these compartments (Supplementary Figure S1a). The PARP1 proximal proteome (FDR < 1%) encompassed 585 proteins that function in RNA biogenesis, cell cycle, DNA replication, and DNA repair (Figure 1B, C, Supplementary Figure S1b, Table S1). We identified previously reported PARP1 interactors that play a role in DNA damage signaling (ATM, MDC1, NBN, RAD50), chromatin remodeling (CHD1L, CHD4, SSRP1, UHRF1), homologous recombination (RPA1, RPA2, BLM, UIMC1) and non-homologous end joining (53BP1, PRKDC, XRCC5/6, LIG1/3) (Figure 1B, D, Supplementary Figure S1c, Table S1). Additionally, we found a group of proteins involved in RNA processing (FUS, AQR, DIS3) and mitosis (TPX2, AURKA, KIF2C, KIF23) (Figure 1B, Supplementary Figure S1c). We further analyzed the PARP1-proximal proteome after inhibition of PARP1 with olaparib to determine which proteins associate with PARP1 in an ADP-ribosylation-dependent manner (Figure 1E, Supplementary Table S2). We identified 185 proteins that dissociated from PARP1 in the presence of olaparib. As reported before, CHD1L was identified in the proximity of PARP1 dependent on ADP-ribosylation (82,83). Moreover, the proximity of proteins involved in DNA replication (ATR, MCM2/3/4/6, RNASEH2A/B, and PCNA) was reduced after inhibition of PARP1 (Figure 1E). On the contrary, proteins involved in mRNA processing (SRSF1, ALYREF and THOC1/3), NHEJ (XRCC5/6), mitosis (TPX2), and BER (APEX1, LIG3) were found proximal to PARP1 independent of its catalytic activity (Figure 1E). To identify proteins that potentially regulate PARP1 activity at stressed replication forks in S-phase, we performed isolation of proteins on nascent DNA (iPOND) (84,85) after inducing replication stress by nucleotide depletion. To this end, we treated U2OS cells for a prolonged time (18 h) with 2 mM hydroxyurea (HU), which leads to replication fork collapse and double-strand breaks (DSBs) in S-phase (Supplementary Figure S2a, Table S3) (84,86). We employed SILAC-based quantitative MS to distinguish proteins that are recruited to unperturbed replication forks and persistently stalled replication forks (2 mM HU, 18 h). We identified 109 proteins that associated with replication forks after persistent replication stress (FDR < 5%) (Supplementary Table S3). DNA repair proteins such as the BRCA1/BARD1 complex, MRE11, MDC1, FANCI, and FANCD2 interacted with replication forks after persistent replication stress (Figure 2A, Supplementary Figure S2b). As previously reported (87), checkpoint protein MDC1 associated less with persistently stalled forks (18 h HU) than with acutely stalled forks (2 h HU) (Supplementary Figure S2c). In addition, RNA processing proteins such as THRAP3, DDX27, DDX39B, ADAR, and subunits of the RNA Pol II complex were identified (Figure 2A, B, Supplementary Figure S2b, c). Notably, several proteins with mitotic functions such as ANLN, TPX2, KIF22, KIF23, KIFC1, and KIF2C were recruited to replication forks after prolonged HU treatment (Figure 2A, B). One of these proteins, Targeting protein for Xklp2 (TPX2), was identified as PARP1 proximal protein that associated with replication forks after persistent replication stress (Figures 1B and 2A, Supplementary Figure S2c). The proximity between TPX2 and PARP1 was independent of the catalytic activity of PARP1 (Figure 1E). During its canonical role, TPX2 interacts with Aurora kinase A and promotes microtubule polymerization in mitosis (58,60,61,88). We confirmed the recruitment of TPX2 to stressed replication forks by iPOND-Western blot analysis (Figure 2C). As a complementary approach, we performed Proximity Ligation Assay (PLA) between TPX2 and single-stranded DNA-binding protein RPA2. Increased proximity between TPX2 and RPA2 was observed after 18 h HU treatment (Figure 2D). Similarly, PARP1 displayed increased proximity to RPA2 after persistent replication stress (Figure 2E).

### Canonical mitotic factors co-occupy stressed replication forks with PARP1

**Figure 3.**
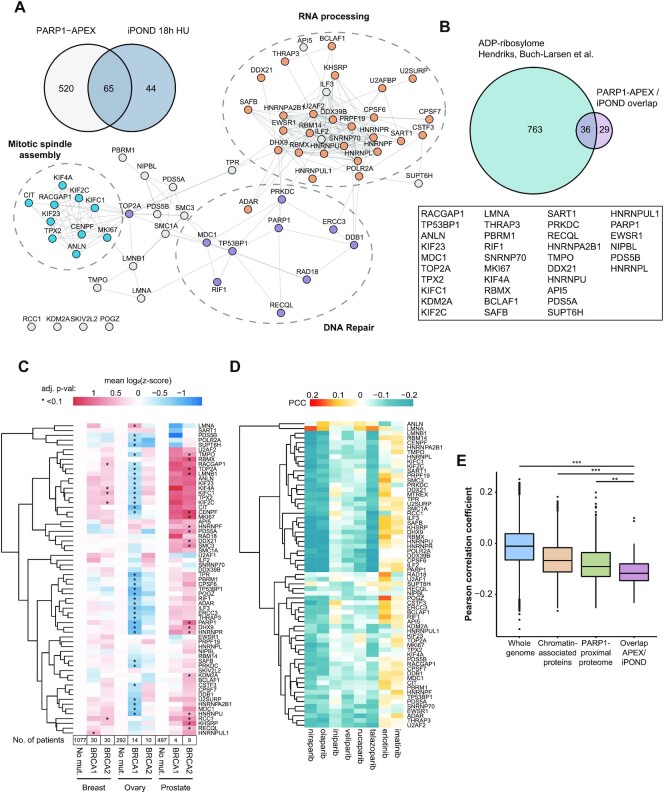
Canonical mitotic factors co-occupy stressed replication forks with PARP1. (**A**) Venn diagram displaying PARP1 proximal proteins that are present at persistently stalled replication forks. Interactions of the 65 proteins from the overlap were mapped in a STRING network using Cytoscape with a 0.7 confidence score cutoff. Mitotic (blue nodes), DNA repair (purple nodes) and RNA processing (orange nodes) clusters are highlighted. (**B**) Number of ADP-ribosylated PARP1 proximal proteins that co-occupy stressed replication forks together with PARP1 based on ADP-ribosylome from Hendriks, Buch-Larsen et al. (8). (**C**) Heatmap of mean z-scored mRNA expression levels in breast, ovarian and prostate cancers extracted from cBioPortal. Multiple t-tests between BRCA1/2-negative and positive cancers were performed to test significance, *adjusted *P*-value <0.1. *P*-values were adjusted using Benjamini-Hochberg correction. Number of patients contributing to the mean expression levels is depicted below each column. (**D**) Heatmap of Pearson correlation coefficient (PCC) between mRNA expression level and sensitivity to indicated drugs based on 558 cancer cell lines from the DepMap portal. Negative correlation indicates reduced cell viability for higher expression levels (blue color). (**E**) Boxplot displaying the Pearson correlation coefficient between mRNA levels and olaparib sensitivity of indicated data sets. Centers of boxplots indicate the median, limits the 25th–75th percentile, whiskers the 10th–90th percentile and dots depict outliers. *P*-values (**P < 0.001, ***P < 0.0001) were derived using *t*-test with Benjamini-Hochberg correction for multiple comparisons.

To identify proteins that occupy collapsed replication forks together with PARP1, we overlapped the PARP1 proximal proteome with proteins enriched at replication forks after persistent replication stress (18 h HU treatment). We focused on the persistent replication stress since in U2OS cells we observed an increased association of PARP1 with replication forks only after 18 h treatment with HU (Figure 2E, Supplementary Figure S2c). The PARP1 proximal proteome and proteins that were associated with stressed replication forks displayed a high interphase chromatin probability (ICP) score (89), confirming that primarily chromatin-associated proteins were identified (Supplementary Figure S3b). Our data highlighted 65 proteins in the PARP1 proximal proteome that were also recruited to replication forks after persistent replication stress, of which 36 have previously been reported to be ADP-ribosylated (Figure 3A, B) (7,90,91). Among those proteins were DNA repair factors such as MDC1 and 53BP1, and RNA processing factors such as THRAP3 and DHX9 (Figure 3A, Supplementary Figure S3b). Interestingly, proteins functioning in cell cycle regulation and mitosis constituted another group of proteins that co-occupied stressed replication forks with PARP1 (Figure 3A, Supplementary Figure S3c). We observed a similar enrichment of mitotic proteins at replication forks after HU treatment for 2 h, indicating that their presence at replication forks is not a consequence of large cell cycle alterations or slippage of cells into mitosis (Supplementary Figure S2c). In addition, TPX2 was also found in the PARP1 proximal proteome in HEK293T cells (log_2_(fold change) = 3.037, *P*-value < 0.01) (Supplementary Figure S3a, Table S2).

Breast, ovarian, and prostate tumors carrying *BRCA1/2* mutations display sensitivity to PARP inhibitors. We hypothesized that HR-deficiency and/or impaired replication fork protection in these tumors might lead to upregulation of proteins that function in DSB repair and replication stress tolerance to compensate for incurred genomic instability. To test this, we extracted gene expression levels for 65 proteins that were proximal to PARP1 and present at stressed replication forks, from breast, ovarian, and prostate cancer samples with wild-type or mutated *BRCA1* or *BRCA2* from the cBioPortal (78). We observed higher mRNA expression levels, especially for the mitotic factors identified at stressed replication forks in breast and prostate cancer patients with *BRCA1/2* mutations compared to the respective *BRCA1/2* wild-type cancers (Figure 3C, Supplementary Figure S3d). Interestingly, lower mRNA expression levels were evident for ovarian cancers with *BRCA1/2* mutations (Figure 3C).

Increased DNA replication stress in cancer cells could lead to an upregulation of pathways that stabilize replication forks, and at the same time, might increase the dependency of these cancer cells on PARP1. Thus, we hypothesized that mRNA levels corresponding to proteins that co-occupy stressed replication forks with PARP1 may serve as a proxy for the dependency of cancer cells on PARP-dependent replication fork protection. To test this hypothesis, we extracted mRNA expression levels of the 65 proteins from 558 cancer cell lines that were treated with PARP inhibitors from the DepMap portal (75,76). We found that increased susceptibility of cancer cell lines to olaparib correlated with higher mRNA expression levels of 65 proteins that co-occupied stressed replication forks with PARP1 (Figure 3D). Interestingly, this trend was significantly more pronounced for the 65 proteins that co-occupied stressed replication forks with PARP1 compared to all chromatin-associated proteins or all PARP1 proximal proteins (Figure 3E, Supplementary Figure S3e). Significant correlations were apparent for the PARP-trapping inhibitors olaparib, niraparib, and talazoparib (Figure 3D). In contrast, low correlations were evident for the non-trapping PARP inhibitors iniparib and veliparib, suggesting that PARP-trapping inhibitors and non-trapping inhibitors display dependencies on distinct protein classes (Figure 3D). The EGFR inhibitor erlotinib and the protein kinase inhibitor imatinib showed opposite correlation patterns compared to the tested PARP inhibitors (Figure 3D).

### TPX2 interacts with DNA repair factors and PARP1

**Figure 4.**
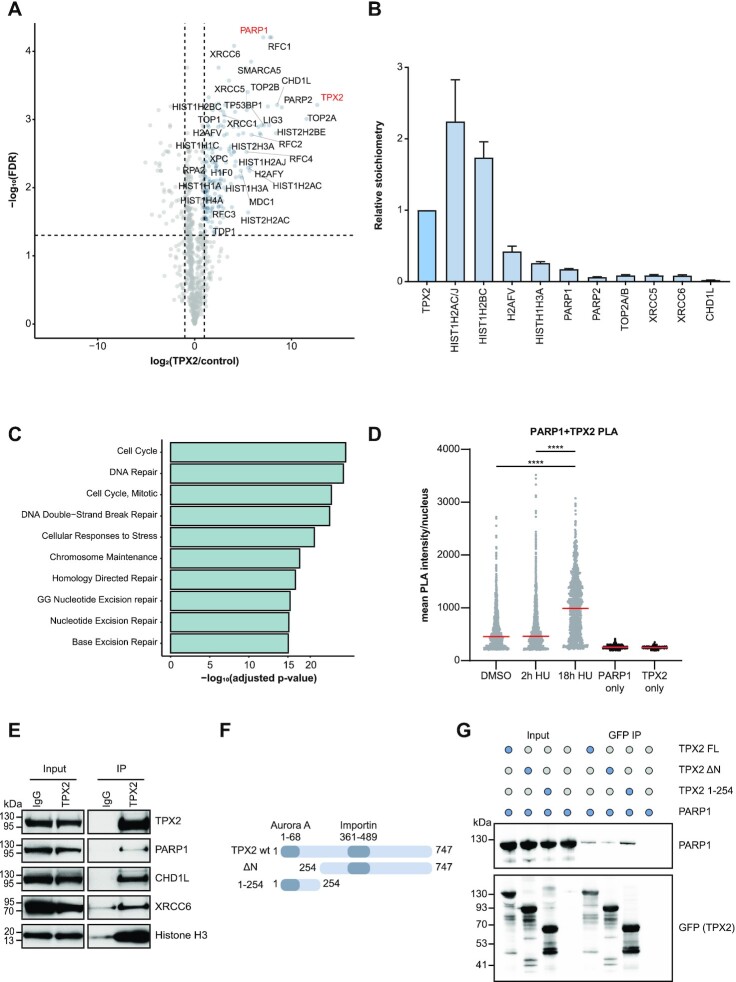
TPX2 interacts with PARP1 and DNA repair factors (**A**) Volcano plot displaying n = 3 biologically independent experiments of LFQ-based interactome of a GFP-tagged TPX2 against a GFP control. The *P*-values were calculated by a two-sample t-test and adjusted for multiple comparisons using the Benjamini-Hochberg correction. Dotted lines indicate fold change >2, <–2 and 5% FDR. (**B**) Relative stoichiometry of the LFQ-based TPX2 interactions. Intensity-based absolute quantification (IBAQ) values for each interactor were normalized to the control IBAQ value. Data are represented as mean ± standard deviation. (**C**) Reactome pathways of TPX2 interacting proteins with log_2_ fold change >2 and FDR <5%. Terms are sorted by their adjusted *P*-value calculated using two-sided Fisher′s exact test with Benjamini–Hochberg correction. (**D**) Proximity ligation assay (PLA) between endogenous PARP1 and TPX2. The grey dots represent the individual values of the mean PLA intensity per nucleus; the red line indicates the median. *P*-values (****P-value < 0.0001) were derived using one-way-ANOVA with Tukey correction for multiple comparisons. (**E**) Western blot validation of indicated TPX2 interactors using an antibody against endogenous TPX2 or an IgG control for immunoprecipitation. (**F**) Schematics of full-length TPX2 and mutants used for pull down experiments. Numbers indicate the respective amino acid positions. Dark shaded regions indicate interaction domain with Aurora A and Importin. (**G**) Western blot analysis of an *in vitro* pull down between recombinant PARP1 and His-GFP-tagged TPX2 full-length protein and its mutants. Blue dots indicate presence of a protein in the respective condition.

To decipher the interaction partners of TPX2, we transiently expressed GFP-tagged TPX2 in U2OS cells and identified co-immunoprecipitated proteins by MS using label-free quantification (LFQ). We confirmed that the pan-nuclear localization of endogenous TPX2 was mirrored by the exogenously expressed GFP-TPX2 (Supplementary Figure S4a). PARP1, PARP2, and CHD1L co-immunoprecipitated with TPX2 demonstrating that TPX2 is not only in proximity but also interacts with PARP1 (Figure 4A). We identified several additional DNA damage response proteins, including MDC1, 53BP1, LIG3, TOP2A and XRCC5/6 as TPX2 interaction partners (Figure 4A, Supplementary Table S4). Additionally, core histones and components of the replication machinery such as RFC2, RFC5 and RPA1 associated with TPX2 (Figure 4A). The interaction of GFP-TPX2 and endogenous PARP1 was confirmed with SILAC-based co-IP-MS and Western blotting (Supplementary Figure S4b, c). Further analysis of the LFQ-based co-IP data revealed that the interaction partners of TPX2 with the highest stoichiometry were histones and PARP1/2 (Figure 4B) (63). Proteins interacting with TPX2 were involved in cell cycle progression and DNA repair pathways such as homology-directed repair, nucleotide excision repair (NER), and base excision repair (BER) (Figure 4C). We confirmed the proximity of endogenous TPX2 and PARP1 in the nucleus by PLAs (Figure 4D). Notably, the proximity of TPX2 and PARP1 was particularly increased after persistent replication stress (18 h HU), suggesting a shared role at persistently stalled replication forks (Figure 4D). Furthermore, the interactions between endogenous TPX2 with PARP1, CHD1L and XRCC6 were validated by co-IP followed by Western blotting (Figure 4E). To map the interaction surface on TPX2 that binds to PARP1, we generated deletion mutants based on previous interaction studies between TPX2 and AURKA (59,61). The unstructured N-terminus of TPX2 mediates the interaction with AURKA, while the centre is predicted to facilitate the binding to importins and the more structured C-terminus is predicted to form alpha-helical repeats (Figure 4F) (61,92). We found that the N-terminal part of TPX2 (1–254 amino acids) was crucial and sufficient for the binding of recombinant PARP1 to recombinant TPX2 *in vitro*, suggesting a direct interaction (Figure 4G, Supplementary Figure S4e). SILAC-based co-IP-MS of the N-terminal deletion mutant or full-length TPX2 in U2OS cells revealed that the N-terminus of TPX2 mediates protein-protein interaction with multiple DNA repair proteins such as PARP1, LIG3, TOP2A and XRCC5/6 (Supplementary Figure S4d). Taken together, our data suggest that PARP1 and TPX2 directly interact via the unstructured N-terminal region (1–254) of TPX2.

### TPX2 promotes ADP-ribosylation of PARP1

**Figure 5.**
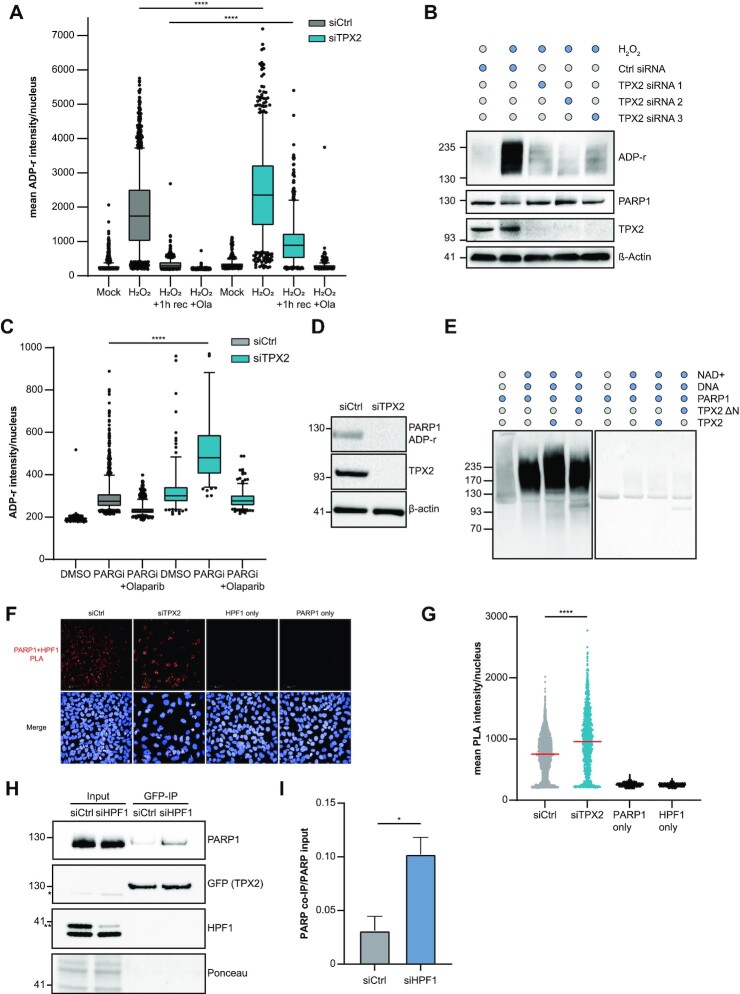
TPX2 modulates PARP1 activity *in vitro* and *in vivo*. (**A**) Representative boxplot of *n* = 2 biologically independent experiments displaying the mean ADP-ribosylation per nucleus after a TPX2 (blue) or a control knockdown (grey) for 24 h. Cells were treated with 2 mM H_2_O_2_ for 10 min ± addition of 1 μM Olaparib and ±1 h recovery. The centres of the boxplots indicate the median, limits the 25th–75th percentile, whiskers the 10th–90th percentile, and dots indicate outliers. ****P-value < 0.0001, one-way ANOVA with Tukey correction for multiple comparisons. (**B**) ADP-ribosylation western blot after TPX2 knockdown in U2OS cells with three individual siRNAs after 2 mM H_2_O_2_ for 10 min. Control cells were pre-treated for 1 h with 1 μM olaparib. (**C**) Representative boxplot of *n* = 2 biologically independent experiments displaying the mean ADP-ribosylation per nucleus after a TPX2 (blue) or a non-targeting control knockdown (grey) for 24 h. Cells were treated with 10 mM PARGi for 1 h ± addition of 1 μM Olaparib. The centres of the boxplots indicate the median, limits the 25th–75th percentile, whiskers the 10th–90th percentile, and dots indicate outliers. ****P-value < 0.0001, one-way ANOVA with Tukey correction for multiple comparisons. (**D**) Western blot displaying endogenous auto-ADP-ribosylation of PARP1 after TPX2 or control knockdown. (**E**) Representative Western blot of *n* = 2 biologically independent experiments of the i*n vitro* ADP-ribosylation assay with purified PARP1 and TPX2 proteins (full-length and ΔN mutant). Staining with an antibody against poly-ADP-ribosylation (left) and Ponceau staining (right). (**F**) Representative images of a proximity ligation assay (PLA) between endogenous HPF1 and PARP1 after knockdown of TPX2 or a control knockdown. PLA signal is displayed in red; Hoechst33342 staining is shown in blue. (**G**) Quantification of a PLA between HPF1 and PARP1 after TPX2 (turquoise) or control knockdown (grey). Individual values of the mean PLA intensity per nucleus are shown; the red line indicates the median. Antibody leave-out controls are shown in black. ****P-value < 0.0001, one-way ANOVA with Tukey correction for multiple comparisons. (**H**) Representative Western blot of the co-immunoprecipitation (IP) of GFP-tagged TPX2 after either a control knockdown or HPF1 knockdown. Inputs are shown on the left, GFP-IP on the right. * indicates the residual PARP1 staining after re-probing with the GFP antibody, while **indicates the specific HPF1 band. (**I**) Bar plot showing the mean ± SD derived from *n* = 2 biologically independent experiments of the GFP-TPX2 co-IP after HPF1 (blue) or control knockdown (grey). *P-value < 0.05, student′s *t*-test.

Based on the finding that TPX2 interacts with PARP1, we tested whether TPX2 regulates the enzymatic activity of PARP1. We depleted TPX2 in U2OS cells using siRNA and monitored changes in the H_2_O_2_-induced nuclear ADP-ribosylation (Figure 5A). Loss of TPX2 led to significantly increased nuclear ADP-ribosylation in response to H_2_O_2_, which persisted after 1 h recovery from oxidative stress (Figure 5A). On the contrary, the knockdown of TPX2 using three individual siRNAs prevented the excessive auto-ADP-ribosylation of PARP1 in response to H_2_O_2_ (Figure 5B). Conversely, overexpression of TPX2 resulted in hyper-ADP-ribosylation of PARP1 in response to oxidative stress (Supplementary Figure S5a). PARP1 has recently been reported to participate in a backup pathway to process un-ligated Okazaki fragments together with XRCC1 and LIG3 (17). Since we identified the interaction of TPX2 with PARP1, XRCC1 and LIG3 (Figure 4A), we further explored the possibility that TPX2 regulates PARP1 during Okazaki fragment ligation. As previously reported (17), PARG inhibition led to the spontaneous accumulation of nuclear ADP-ribosylation specifically in S-phase cells (Supplementary Figure S5b). Similar to the H_2_O_2_ treatment, we found significantly increased nuclear ADP-ribosylation in TPX2 knockdown cells after PARG inhibition (Figure 5C). Moreover, we observed endogenous auto-modification of PARP1, likely resulting from PARP1 activity during Okazaki fragment processing, which was lost upon TPX2 knockdown (Figure 5D). To test whether TPX2 directly stimulates the catalytic activity of PARP1, we performed *in vitro* ADP-ribosylation assays using recombinant PARP1 and TPX2. We observed *in vitro* ADP-ribosylation activity of purified PARP1 in the presence of DNA and NAD^+^ (Figure 5E). The addition of full-length TPX2 promoted PARP1′s auto-ADP-ribosylation activity, while the N-terminal deletion mutant of TPX2, which lacks the PARP1 binding site, failed to stimulate PARP1 (Figure 5E, Supplementary Figure S5c). In contrast to the observed hypo-auto-modification of PARP1 after TPX2 knockdown, loss of the PARP1 regulator HPF1 leads to hyper-auto-modification of PARP1 and a shift towards serine histone ADP-ribosylation (10–13). As previously reported, recombinant HPF1 promoted the ADP-ribosylation of an H3 peptide *in vitro* (Supplementary Figure S5d), while the knockdown of HPF1 led to hyper-ADP-ribosylated PARP1 *in vivo* (Supplementary Figure S5e). We observed that the addition of recombinant TPX2 reversed the HPF1-dependent switch towards H3 ADP-ribosylation (Supplementary Figure S5d). These findings suggest that TPX2 competes with HPF1 for PARP1 binding to promote auto-modification of PARP1 while preventing ADP-ribosylation of H3. In support of this, we observed a significantly increased PLA signal between PARP1 and HPF1 in TPX2 knockdown cells (Figure 5F, G) as well as increased interaction of PARP1 and TPX2 in HPF1 knockdown cells (Figure 5H, I).

### TPX2 opposes replication stress and promotes DSB repair via HR

**Figure 6.**
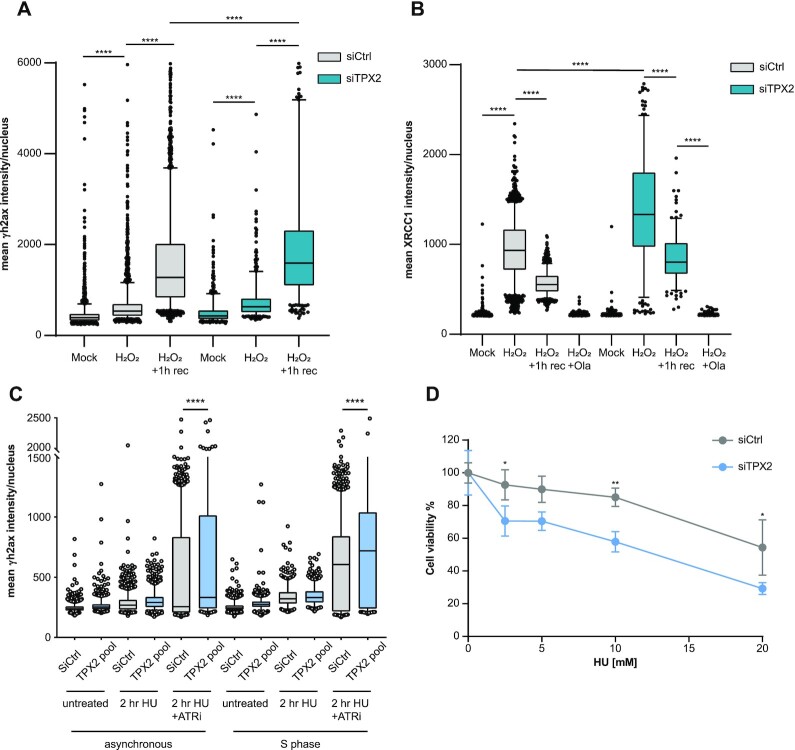
TPX2 promotes HR and opposes S phase specific DSBs (**A**) Immunofluorescence analysis of γH2AX in control (grey) or TPX2 (turquoise) knockdown U2OS cells after treatment with 2 mM H_2_O_2_ for 10 min with or without 1 h recovery. Representative box plot of *n* = 2 biologically independent experiments displaying the quantification of the mean γH2AX intensity per nucleus. Center of boxplots indicate the median, limits the 25th–75th percentile, whiskers the 10th–90th percentile and dots indicate outliers. ****P-value < 0.0001, one-way ANOVA with Tukey correction for multiple comparisons. (**B**) Immunofluorescence analysis of chromatin-bound XRCC1 (under pre-extraction conditions) in control (grey) or TPX2 (turquoise) knockdown U2OS cells after treatment with 2 mM H_2_O_2_ for 10 min with or without 1 h recovery. Representative box plot of *n* = 2 biologically independent experiments displaying the quantification of the mean γH2AX intensity per nucleus. Center of boxplots indicate the median, limits the 25th–75th percentile, whiskers the 10th–90th percentile and dots indicate outliers. ****P-value < 0.0001, one-way ANOVA with Tukey correction for multiple comparisons. (**C**) Immunofluorescence analysis of γH2AX in U2OS cells after treatment with 3 mM HU for 2 h in the presence or absence of ATR inhibitor (4 μM, 1 h). S phase synchronized cells were pre-treated for 18 h with 200 μM l-mimosine and subsequently released into S phase. Asynchronous or S phase synchronized cells were stained 48 h after indicated knockdowns. Boxplot displaying the quantification of the mean γH2AX intensity per nucleus. Center of boxplots indicate the median, limits the 25th–75th percentile, whiskers the 10th–90th percentile and dots indicate outliers. ****P-value < 0.0001, one-way ANOVA with Tukey correction for multiple comparisons. (**D**) Representative cell titer blue assay of *n* = 3 biologically independent experiments, displaying the viability of U2OS cells after control (grey) or TPX2 (blue) knockdown in response to a titration of hydroxyurea (HU). **P-value < 0.01,* *P*-value < 0.05, two-way ANOVA with Sidak correction for multiple comparisons.

Differential ADP-ribosylation levels in TPX2 knockdown cells in response to H_2_O_2_ treatment and interaction of TPX2 with BER proteins led us to investigate whether TPX2 regulates the repair of oxidative stress-induced SSBs. We observed increased γH2AX formation and increased retention of XRCC1 on chromatin in TPX2 knockdown cells after treatment with H_2_O_2_, suggesting that regulation of PARP1 by TPX2 is required for efficient BER (Figure 6A, B). Since we observed the recruitment of TPX2 alongside PARP1 to persistently stalled replication forks, we further tested whether TPX2 is required for the repair of replication-dependent DNA damage. Indeed, we found increased γH2AX and pRPA2 (Ser4/8) levels in TPX2 knockdown cells after treatment with HU and ATRi/HU, particularly in a population of cells that had been synchronized in G1 and subsequently released into S-phase in the presence of the respective inhibitors (Figure 6C, Supplementary Figure S6a). Accordingly, TPX2 knockdown cells displayed increased sensitivity to persistent HU treatment compared to wild-type cells (Figure 6D). Moreover, we found increased replication-dependent DSB formation after loss of TPX2 and treatment with the topoisomerase 1 inhibitor camptothecin (CPT) by neutral comet assay (Supplementary Figure S6b, c). DSBs persisted in TPX2 knockdown cells after 6 h of recovery from CPT, while control cells completed DNA repair (Supplementary Figure S6a, b). The interaction of TPX2 with HR proteins and the observation that *TPX2* mRNA levels are upregulated in breast and prostate cancers with *BRCA1/2* mutations (Supplementary Figure S3d), suggested a role of TPX2 in the repair of DSBs arising from collapsed replication forks by HR. To test whether TPX2 promotes HR, we monitored the efficiency of I-SceI-induced DSB repair through HR by flow cytometry (79). Knockdown of TPX2 led to a significant decrease in HR efficiency (Supplementary Figure S6d). Collectively, these findings suggest that in addition to its canonical function in mitosis, TPX2 regulates PARP1 activity in response to oxidative stress and during perturbed S-phase.

## DISCUSSION

PARP inhibitors are used for the treatment of breast, ovarian, and prostate cancers (33,34). Part of the vulnerability to PARP inhibitors can be accounted to PARP trapping at stalled replication forks and subsequent lack of fork protection in *BRCA*-deficient cells (30,93,94). Moreover, single-strand DNA gap exposure has been proposed as a determining trait of BRCAness and can contribute to the prediction of sensitivity or resistance to PARP inhibitors (21). However, the mechanisms that regulate PARP1 activity in response to replication stress and during replication gap suppression remain poorly characterized. We investigated which proteins co-occupy stressed replication forks together with PARP1 and thus regulate its activity in response to replication stress. We employed quantitative MS to map the PARP1 proximal proteome on native chromatin. Furthermore, we identified proteins that display proximity to PARP1 in a PAR-dependent manner, among which were DNA replication factors such as members of the MCM and the RNaseH2 complexes. VCP and components of the proteasome, which have been reported to remove trapped PARP1 from chromatin after MMS treatment (95) were also proximal to PARP1 in a PAR-dependent manner. Among 109 proteins that were recruited to persistently stalled replication forks, we found core replication fork protection proteins such as MDC1, FANCD2 and the BRCA1/BARD1 complex in line with a previous study conducted in HEK293T cells (87). We identified additional DNA repair proteins (RAD18, MRE11, PARP1 and RECQL) in U2OS cells, while we did not find the cell cycle checkpoint proteins ATR, ATRIP, and ETAA1. Of note, ETAA1 is known to be expressed at very low levels in U2OS cells (96). The observed differences in stressed replisome proteome in U2OS and HEK293T cells are thus likely due to cell line-specific protein expression levels and recruitment patterns as well as technical differences such as the amount of input protein material. We found that 65 of the PARP1 proximal proteins occupied persistently stressed replication forks in U2OS cells, suggesting that they play a role in the cellular response to replication stress together with PARP1. Notably, high mRNA expression levels of PARP1 proximal proteins that are recruited to stressed replication forks correlated with the sensitivity of cancer cells to PARP-trapping inhibitors but not to non-trapping inhibitors. These findings suggest that not only the lack of PARP1 activity but also replication stress induced by PARP trapping contributes to cancer cell sensitivity. Moreover, cancer cells that are sensitive to non-trapping inhibitors and cells that are sensitive to PARP-trapping inhibitors seem to display dependencies on distinct groups of proteins.

Among the proteins that co-occupy stressed replication forks together with PARP1, we identified a cluster of proteins with previously described mitotic functions. While the identified mitotic proteins have not been found in a previous HU-based iPOND study in HEK293T cells (87), nascent chromatin capture proteomics in HeLa cells after HU treatment confirms the association of members of the mitotic cluster (TPX2, KIF22, KIF23, KIF2C and ANLN) to stalled replication forks (97).

One protein of the mitotic cluster, TPX2, associates with AURKA during mitosis and promotes microtubule nucleation and stability (60,61,88,98). The previously reported increased γH2AX foci formation upon γ-irradiation in TPX2 knockdown cells and the presence of TPX2 in γ-irradiation-induced repair foci suggested a role of TPX2 as a DNA damage response protein (98). Expression levels of *TPX2* are increased in a variety of cancers (99–102) and we found that *TPX2* gene expression levels are elevated in breast and ovarian cancers with pathogenic *BRCA* mutations compared to *BRCA* wild-type cancers. In line with the idea that cancer cells with pathogenic *BRCA* mutations depend on TPX2, knockdown of TPX2 has been reported to lead to HR defects and reduced viability of *BRCA2*-deficient cancer cells (103).

Our data demonstrate that TPX2 interacts with PARP1, histones, DNA replication, and DNA repair proteins, providing further evidence that TPX2 functions as a genome stability factor also outside of mitosis. We found that TPX2 associates with PARP1 independent of its ADP-ribosylation activity, and that interaction is increased after persistent replication stress, suggesting TPX2’s function in opposing replication stress. Further biochemical characterization revealed that TPX2 directly interacts with PARP1 via its unstructured N-terminal domain. In addition, we found that the N-terminus of TPX2 is crucial to promote the auto-ADP-ribosylation activity of PARP1 *in vitro*, suggesting that binding of TPX2 is required to promote the auto-modification of PARP1 *in vivo*. Notably, TPX2-dependent regulation of PARP1 is important for efficient BER since hypo-auto-modification of PARP1 in TPX2 knockdown cells in response to H_2_O_2_ is accompanied by reduced SSB repair efficiency and increased chromatin retention of XRCC1. We propose that TPX2 and HPF1 compete for PARP1 binding, thereby fine-tuning the catalytic activity and the downstream targets of PARP1. Accordingly, we found an increased association of HPF1 and PARP1 in TPX2 knockdown cells accompanied by hypo-modification of PARP1 and an increased association of TPX2 and PARP1 in HPF1 knockdown cells accompanied by hyper-modification of PARP1. While TPX2 knockdown led to reduced auto-ADP-ribosylation of PARP1, we observed an increase in total nuclear ADP-ribosylation upon oxidative stress. This might be a consequence of a stronger association of PARP1 and HPF1 that results in increased HPF1-dependent serine ADP-ribosylation of histones and other proteins. Taken together, these findings suggest that TPX2 promotes auto-ADP-ribosylation of PARP1 while inhibiting HPF1-dependent targeting towards serine residues. Thus, we propose that TPX2 constitutes an additional switch to HPF1 that fine-tunes the catalytic activity and substrate specificity of PARP1. HPF1-dependent auto-modification of PARP1 on Ser499, Ser507 and Ser519 has been described as a mechanism to reduce PARP1 trapping on chromatin, thus increasing PARP inhibitor tolerance (15). Since TPX2 and HPF1 displayed opposite regulatory effects on PARP1, TPX2 might instead increase PARP1 trapping on chromatin. Notably, overexpression of TPX2 led to increased PARP1 activity and thus to increased PARP trapping potential, providing an explanation for why high *TPX2* mRNA levels in cancer cells correlate with increased sensitivity to PARP trapping inhibitors.

Previous MS-based studies identified multiple ADP-ribosylation sites on TPX2 after treatment of human cells with H_2_O_2_ and MMS . The ADP-ribosylation sites are located within or very proximal to the N-terminus of TPX2, thus supporting our finding that this region mediates the interaction with PARP1. The finding that TPX2 and PARP1 interact constitutively, in the absence of ADP-ribosylation on TPX2, suggests a DNA damage-independent function of the TPX2-PARP1 interaction. During replication, PARP1 acts as a sensor of un-ligated Okazaki fragments and promotes their ligation in a LIG3-XRCC1-dependent manner, distinct from the canonical LIG1-dependent pathway (17). In this study, we identified LIG3 and XRCC1 as interaction partners of TPX2. In the presence of PARG inhibitors, we observed increased nuclear ADP-ribosylation levels in TPX2 knockdown cells in S-phase, suggesting defects during lagging strand synthesis (17). Similar to the auto-modification of PARP1 in TPX2 knockdown cells in response to H_2_O_2_, we found decreased endogenous auto-modification of PARP1 after TPX2 depletion. Our data suggest that TPX2-dependent regulation of PARP1 is important for faithful non-canonical Okazaki fragment processing. Strikingly, loss of *BRCA1/2* leads to exposure of replication gaps due to aberrant lagging strand synthesis, which is suppressed by the PARP1–LIG3–XRCC1 axis (21). Therefore, the dependency of *BRCA*-deficient cells on TPX2 might result from its role in PARP1 regulation during replication gap suppression. Moreover, TPX2 functions in a distinct fork protection pathway from BRCA1, by counteracting 53BP1-dependent fork degradation (104). In line with these findings, we observed that TPX2 protects cells against S-phase specific DNA damage and that loss of TPX2 results in significantly reduced HR-dependent DNA DSB repair and sensitivity to HU. Since PARP1 binds directly to the N-terminal domain of TPX2, which also serves as the interaction surface for AURKA, the role of TPX2 in PARP1 regulation is likely independent of the associated kinase.

## DATA AVAILABILITY

The mass spectrometry-based proteomics data have been deposited to the ProteomeXchange Consortium via the PRIDE partner repository (105) with the dataset identifier PXD037154 (https://www.ebi.ac.uk/pride/archive/projects/ PXD037154).

## Supplementary Material

gkac948_Supplemental_Files

## References

[1] Eleazer R. , Fondufe-Mittendorf Y.N. The multifaceted role of PARP1 in RNA biogenesis. Wiley Interdiscip. Rev. RNA. 2021; 12:e1617. 32656996 10.1002/wrna.1617 PMC7856298

[2] Chaudhuri A.R. , Nussenzweig A. The multifaceted roles of PARP1 in DNA repair and chromatin remodelling. Nat. Rev. Mol. Cell Biol. 2017; 18:610–621. 28676700 10.1038/nrm.2017.53 PMC6591728

[3] Wei H. , Yu X. Functions of PARylation in DNA damage repair pathways. Genomics, Proteomics & Bioinformatics. 2016; 14:131–139. 10.1016/j.gpb.2016.05.001 PMC4936651 27240471

[4] Chatterjee N. , Walker G.C. Mechanisms of DNA damage, repair and mutagenesis. Environ. Mol. Mutagen. 2017; 58:235–263. 28485537 10.1002/em.22087 PMC5474181

[5] Khodyreva S.N. , Lavrik O.I. Poly(ADP-Ribose) polymerase 1 as a key regulator of DNA repair. Mol. Biol. 2016; 50:580–595. 10.7868/S0026898416040030 27668604

[6] Jungmichel S. , Rosenthal F., Altmeyer M., Lukas J., Hottiger M.O., Nielsen M.L. Proteome-wide identification of poly(ADP-Ribosyl)ation targets in different genotoxic stress responses. Mol. Cell. 2013; 52:272–285. 24055347 10.1016/j.molcel.2013.08.026

[7] Hendriks I.A. , Buch-Larsen S.C., Prokhorova E., Elsborg J.D., Rebak A.K.L.F.S., Zhu K., Ahel D., Lukas C., Ahel I., Nielsen M.L. The regulatory landscape of the human HPF1- and ARH3-dependent ADP-ribosylome. Nat. Commun. 2021; 12:5893. 34625544 10.1038/s41467-021-26172-4 PMC8501107

[8] Buch-Larsen S.C. , Rebak A.K.L.F.S., Hendriks I.A., Nielsen M.L. Temporal and site-specific ADP-Ribosylation dynamics upon different genotoxic stresses. Cells. 2021; 10:2927. 34831150 10.3390/cells10112927 PMC8616546

[9] Martello R. , Leutert M., Jungmichel S., Bilan V., Larsen S.C., Young C., Hottiger M.O., Nielsen M.L. Proteome-wide identification of the endogenous ADP-ribosylome of mammalian cells and tissue. Nat. Commun. 2016; 7:12917. 27686526 10.1038/ncomms12917 PMC5056437

[10] Gibbs-Seymour I. , Fontana P., Rack J.G.M., Ahel I. HPF1/C4orf27 is a PARP-1-Interacting protein that regulates PARP-1 ADP-Ribosylation activity. Mol. Cell. 2016; 62:432–442. 27067600 10.1016/j.molcel.2016.03.008 PMC4858568

[11] Bonfiglio J.J. , Fontana P., Zhang Q., Colby T., Gibbs-seymour I., Atanassov I., Bartlett E., Zaja R., Ahel I., Matic I. Serine ADP-ribosylation depends on HPF1. Mol. Cell. 2017; 65:932–940. 28190768 10.1016/j.molcel.2017.01.003 PMC5344681

[12] Suskiewicz M.J. , Zobel F., Ogden T.E.H., Fontana P., Ariza A., Yang J.-C., Zhu K., Bracken L., Hawthorne W.J., Ahel D. et al. HPF1 completes the PARP active site for DNA damage-induced ADP-ribosylation. Nature. 2020; 579:598–602. 32028527 10.1038/s41586-020-2013-6 PMC7104379

[13] Palazzo L. , Leidecker O., Prokhorova E., Dauben H., Matic I., Ahel I. Serine is the major residue for ADP- ribosylation upon DNA damage. Elife. 2018; 7:e34334. 29480802 10.7554/eLife.34334 PMC5837557

[14] Fontana P. , Bonfiglio J.J., Palazzo L., Bartlett E., Matic I., Ahel I. Serine ADP-ribosylation reversal by the hydrolase ARH3. Elife. 2017; 6:e28533. 28650317 10.7554/eLife.28533 PMC5552275

[15] Prokhorova E. , Zobel F., Smith R., Zentout S., Gibbs-Seymour I., Schützenhofer K., Peters A., Groslambert J., Zorzini V., Agnew T. et al. Serine-linked PARP1 auto-modification controls PARP inhibitor response. Nat. Commun. 2021; 12:4055. 34210965 10.1038/s41467-021-24361-9 PMC8249464

[16] Ying S. , Hamdy F.C., Helleday T. Mre11-dependent degradation of stalled DNA replication forks is prevented by BRCA2 and PARP1. Cancer Res. 2012; 72:2814–2821. 22447567 10.1158/0008-5472.CAN-11-3417

[17] Hanzlikova H. , Kalasova I., Demin A.A., Pennicott L.E., Cihlarova Z., Caldecott K.W. The importance of poly(adp-ribose) polymerase as a sensor of unligated okazaki fragments during DNA replication. Mol. Cell. 2018; 71:319–331. 29983321 10.1016/j.molcel.2018.06.004 PMC6060609

[18] Bryant H.E. , Petermann E., Schultz N., Jemth A.S., Loseva O., Issaeva N., Johansson F., Fernandez S., McGlynn P., Helleday T. PARP is activated at stalled forks to mediate Mre11-dependent replication restart and recombination. EMBO J. 2009; 28:2601–2615. 19629035 10.1038/emboj.2009.206 PMC2738702

[19] Ronson G.E. , Piberger A.L., Higgs M.R., Olsen A.L., Stewart G.S., McHugh P.J., Petermann E., Lakin N.D. PARP1 and PARP2 stabilise replication forks at base excision repair intermediates through Fbh1-dependent rad51 regulation. Nat. Commun. 2018; 9:746. 29467415 10.1038/s41467-018-03159-2 PMC5821833

[20] Ray Chaudhuri A. , Hashimoto Y., Herrador R., Neelsen K.J., Fachinetti D., Bermejo R., Cocito A., Costanzo V., Lopes M. Topoisomerase i poisoning results in PARP-mediated replication fork reversal. Nat. Struct. Mol. Biol. 2012; 19:417–423. 22388737 10.1038/nsmb.2258

[21] Cong K. , Peng M., Kousholt A.N., Lee W.T.C., Lee S., Nayak S., Krais J., VanderVere-Carozza P.S., Pawelczak K.S., Calvo J. et al. Replication gaps are a key determinant of PARP inhibitor synthetic lethality with BRCA deficiency. Mol. Cell. 2021; 81:3128–3144. 34216544 10.1016/j.molcel.2021.06.011 PMC9089372

[22] Maya-Mendoza A. , Moudry P., Merchut-Maya J.M., Lee M., Strauss R., Bartek J. High speed of fork progression induces DNA replication stress and genomic instability. Nature. 2018; 559:279–284. 29950726 10.1038/s41586-018-0261-5

[23] Genois M.-M. , Gagné J.-P., Yasuhara T., Jackson J., Saxena S., Langelier M.-F., Ahel I., Bedford M.T., Pascal J.M., Vindigni A. et al. CARM1 regulates replication fork speed and stress response by stimulating PARP1. Mol. Cell. 2021; 81:784–800. 33412112 10.1016/j.molcel.2020.12.010 PMC7897296

[24] Panzarino N.J. , Krais J.J., Cong K., Peng M., Mosqueda M., Nayak S.U., Bond S.M., Calvo J.A., Doshi M.B., Bere M. et al. Replication gaps underlie BRCA deficiency and therapy response. Cancer Res. 2021; 81:1388–1397. 33184108 10.1158/0008-5472.CAN-20-1602 PMC8026497

[25] Ceccaldi R. , Rondinelli B., D ’andrea A.D. Repair pathway choices and consequences at the Double- Strand Break mechanisms of DNA DSB repair. Trends Biochem. Sci. 2016; 26:52–64. 10.1016/j.tcb.2015.07.009 PMC4862604 26437586

[26] Daley J.M. , Sung P. 53BP1, BRCA1, and the choice between recombination and end joining at DNA double-strand breaks. Mol Cell Biol. 2014; 34:1380–1388. 24469398 10.1128/MCB.01639-13 PMC3993578

[27] Scully R. , Panday A., Elango R. DNA double strand break repair pathway choice in somatic mammalian cells. Nat. Rev. Mol. Cell. 2019; 20:698–714. 10.1038/s41580-019-0152-0 PMC7315405 31263220

[28] Quinet A. , Lemaçon D., Vindigni A. Replication fork reversal: players and guardians. Mol. Cell. 2017; 68:830–833. 29220651 10.1016/j.molcel.2017.11.022 PMC5895179

[29] O’Connor M.J. Targeting the DNA damage response in cancer. Mol. Cell. 2015; 60:547–560. 26590714 10.1016/j.molcel.2015.10.040

[30] Chaudhuri A.R. , Callen E., Ding X., Gogola E., Duarte A.A., Lee J.E., Wong N., Lafarga V., Calvo J.A., Panzarino N.J. et al. Replication fork stability confers chemoresistance in BRCA-deficient cells. Nature. 2016; 535:382–387. 27443740 10.1038/nature18325 PMC4959813

[31] Farmer H. , McCabe H., Lord C.J., Tutt A.H.J., Johnson D.A., Richardson T.B., Santarosa M., Dillon K.J., Hickson I., Knights C. et al. Targeting the DNA repair defect in BRCA mutant cells as a therapeutic strategy. Nature. 2005; 434:917–921. 15829967 10.1038/nature03445

[32] Bryant H.E. , Schultz N., Thomas H.D., Parker K.M., Flower D., Lopez E., Kyle S., Meuth M., Curtin N.J., Helleday T. Specific killing of BRCA2-deficient tumours with inhibitors of poly(ADP-ribose) polymerase. Nature. 2005; 434:913–917. 15829966 10.1038/nature03443

[33] Helleday T. The underlying mechanism for the PARP and BRCA synthetic lethality: clearing up the misunderstandings. Mol. Oncol. 2011; 5:387–393. 21821475 10.1016/j.molonc.2011.07.001 PMC5528309

[34] Lord C.J. , Ashworth A. PARP inhibitors: synthetic lethality in the clinic. Science. 2017; 355:1152–1158. 28302823 10.1126/science.aam7344 PMC6175050

[35] Kuchenbaecker K.B. , Hopper J.L., Barnes D.R., Phillips K.A., Mooij T.M., Roos-Blom M.J., Jervis S., Van Leeuwen F.E., Milne R.L., Andrieu N. et al. Risks of breast, ovarian, and contralateral breast cancer for BRCA1 and BRCA2 mutation carriers. J. Am. Med. Assoc. 2017; 317:2402–2416. 10.1001/jama.2017.7112 28632866

[36] Oh M. , Alkhushaym N., Fallatah S., Althagafi A., Aljadeed R., Alsowaida Y., Jeter J., Martin J.R., Babiker H.M., McBride A. et al. The association of BRCA1 and BRCA2 mutations with prostate cancer risk, frequency, and mortality: A meta-analysis. Prostate. 2019; 79:880–895. 30900310 10.1002/pros.23795

[37] Nyberg T. , Frost D., Barrowdale D., Evans D.G., Bancroft E., Adlard J., Ahmed M., Barwell J., Brady A.F., Brewer C. et al. Prostate cancer risks for male BRCA1 and BRCA2 mutation carriers: a prospective cohort study. Eur. Urol. 2020; 77:24–35. 31495749 10.1016/j.eururo.2019.08.025 PMC6926480

[38] Levy-Lahad E. , Friedman E. Cancer risks among BRCA1 and BRCA2 mutation carriers. Br. J. Cancer. 2007; 96:11–15. 17213823 10.1038/sj.bjc.6603535 PMC2360226

[39] Manchana T. , Phoolcharoen N., Tantbirojn P. BRCA mutation in high grade epithelial ovarian cancers. Gynecol. Oncol. 2019; 29:102–105. 10.1016/j.gore.2019.07.007 PMC6710551 31467961

[40] Alsop K. , Fereday S., Meldrum C., DeFazio A., Emmanuel C., George J., Dobrovic A., Birrer M.J., Webb P.M., Stewart C. et al. BRCA mutation frequency and patterns of treatment response in BRCA mutation-positive women with ovarian cancer: a report from the australian ovarian cancer study group. J. Clin. Oncol. 2012; 30:2654–2663. 22711857 10.1200/JCO.2011.39.8545 PMC3413277

[41] Armstrong N. , Ryder S., Forbes C., Ross J., Quek R.G.W. A systematic review of the international prevalence of BRCA mutation in breast cancer. Clin. Epidemiol. 2019; 11:543–561. 31372057 10.2147/CLEP.S206949 PMC6628947

[42] Kim H. , Cho D.-Y., Choi D.H., Choi S.-Y., Shin I., Park W., Huh S.J., Han S.-H., Lee M.H., Ahn S.H. et al. Characteristics and spectrum of BRCA1 and BRCA2 mutations in 3, 922 korean patients with breast and ovarian cancer. Breast Cancer Res. Treat. 2012; 134:1315–1326. 22798144 10.1007/s10549-012-2159-5

[43] Palomba G. , Budroni M., Olmeo N., Atzori F., Ionta M.T., Pisano M., Tanda F., Cossu A., Palmieri G. Triple-negative breast cancer frequency and type of BRCA mutation: clues from sardinia. Oncol. Lett. 2014; 7:948–952. 24944648 10.3892/ol.2014.1834 PMC3961447

[44] Patel A.G. , Sarkaria J.N., Kaufmann S.H. Nonhomologous end joining drives poly(ADP-ribose) polymerase (PARP) inhibitor lethality in homologous recombination-deficient cells. Proc. Natl. Acad. Sci. U.S.A. 2011; 108:3406–3411. 21300883 10.1073/pnas.1013715108 PMC3044391

[45] Li H. , Liu Z.Y., Wu N., Chen Y.C., Cheng Q., Wang J. PARP inhibitor resistance: the underlying mechanisms and clinical implications. Mol. Cancer. 2020; 19:107. 32563252 10.1186/s12943-020-01227-0 PMC7305609

[46] Fong P.C. , Yap T.A., Boss D.S., Carden C.P., Mergui-Roelvink M., Gourley C., De Greve J., Lubinski J., Shanley S., Messiou C. et al. Poly(ADP)-ribose polymerase inhibition: frequent durable responses in BRCA carrier ovarian cancer correlating with platinum-free interval. J. Clin. Oncol. 2010; 28:2512–2519. 20406929 10.1200/JCO.2009.26.9589

[47] Rondinelli B. , Gogola E., Yücel H., Duarte A.A., Van De Ven M., Van Der Sluijs R., Konstantinopoulos P.A., Jonkers J., Ceccaldi R., Rottenberg S. et al. EZH2 promotes degradation of stalled replication forks by recruiting MUS81 through histone H3 trimethylation. Nat. Cell Biol. 2017; 19:1371–1378. 29035360 10.1038/ncb3626

[48] Pettitt S.J. , Krastev D.B., Brandsma I., Dréan A., Song F., Aleksandrov R., Harrell M.I., Menon M., Brough R., Campbell J. et al. Genome-wide and high-density CRISPR-Cas9 screens identify point mutations in PARP1 causing PARP inhibitor resistance. Nat. Commun. 2018; 9:1849. 29748565 10.1038/s41467-018-03917-2 PMC5945626

[49] Gogola E. , Duarte A.A., de Ruiter J.R., Wiegant W.W., Schmid J.A., de Bruijn R., James D.I., Guerrero Llobet S., Vis D.J., Annunziato S. et al. Selective loss of PARG restores PARylation and counteracts PARP inhibitor-mediated synthetic lethality. Cancer Cell. 2018; 33:1078–1093. 29894693 10.1016/j.ccell.2018.05.008

[50] Michelena J. , Lezaja A., Teloni F., Schmid T., Imhof R., Altmeyer M. Analysis of PARP inhibitor toxicity by multidimensional fluorescence microscopy reveals mechanisms of sensitivity and resistance. Nat. Commun. 2018; 9:2678. 29992957 10.1038/s41467-018-05031-9 PMC6041334

[51] Sakai W. , Swisher E.M., Karlan B.Y., Agarwal M.K., Higgins J., Friedman C., Villegas E., Jacquemont C., Farrugia D.J., Couch F.J. et al. Secondary mutations as a mechanism of cisplatin resistance in BRCA2-mutated cancers. Nature. 2008; 451:1116–1120. 18264087 10.1038/nature06633 PMC2577037

[52] Edwards S.L. , Brough R., Lord C.J., Natrajan R., Vatcheva R., Levine D.A., Boyd J., Reis-Filho J.S., Ashworth A. Resistance to therapy caused by intragenic deletion in BRCA2. Nature. 2008; 451:1111–1115. 18264088 10.1038/nature06548

[53] Johnson N. , Johnson S.F., Yao W., Li Y.C., Choi Y.E., Bernhardy A.J., Wang Y., Capelletti M., Sarosiek K.A., Moreau L.A. et al. Stabilization of mutant BRCA1 protein confers PARP inhibitor and platinum resistance. Proc. Natl. Acad. Sci. U.S.A. 2013; 110:17041–17046. 24085845 10.1073/pnas.1305170110 PMC3801063

[54] Wang Y. , Bernhardy A.J., Cruz C., Krais J.J., Nacson J., Nicolas E., Peri S., van der Gulden H., van der Heijden I., O′Brien S.W. et al. The BRCA1- 11q alternative splice isoform bypasses germline mutations and promotes therapeutic resistance to PARP inhibition and cisplatin. Cancer Res. 2017; 76:2778–2790. 10.1158/0008-5472.CAN-16-0186 PMC4874568 27197267

[55] Jaspers J.E. , Kersbergen A., Boon U., Sol W., van Deemter L., Zander S.A., Drost R., Wientjens E., Ji J., Aly A. et al. Loss of 53BP1 causes PARP inhibitor resistance in Brca1- Mutated Mouse mammary tumors. Cancer Discov. 2013; 3:68–81. 23103855 10.1158/2159-8290.CD-12-0049 PMC7518105

[56] Noordermeer S.M. , van Attikum H. PARP inhibitor resistance: a Tug-of-War in BRCA-Mutated cells. Trends Cell Biol. 2019; 29:820–834. 31421928 10.1016/j.tcb.2019.07.008

[57] Lam S.S. , Martell J.D., Kamer K.J., Deerinck T.J., Ellisman M.H., Mootha V.K., Ting A.Y. Directed evolution of APEX2 for electron microscopy and proteomics. Nat. Methods. 2015; 12:51–54. 25419960 10.1038/nmeth.3179 PMC4296904

[58] Gruss O.J. , Carazo-Salas R.E., Schatz C.A., Guarguaglini G., Kast J., Wilm M., Le Bot N., Vernos I., Karsenti E., Mattaj I.W. Ran induces spindle assembly by reversing the inhibitory effect of importin α on TPX2 activity. Cell. 2001; 104:83–93. 11163242 10.1016/s0092-8674(01)00193-3

[59] Kufer T.A. , Silljé H.H.W., Körner R., Gruss O.J., Meraldi P., Nigg E.A. Human TPX2 is required for targeting Aurora-A kinase to the spindle. J. Cell Biol. 2002; 158:617–623. 12177045 10.1083/jcb.200204155 PMC2174010

[60] Tsai M.-Y. , Wiese C., Cao K., Martin O., Donovan P., Ruderman J., Prigent C., Zheng Y. A ran signalling pathway mediated by the mitotic kinase aurora a in spindle assembly. Nat. Cell Biol. 2003; 5:242–248. 12577065 10.1038/ncb936

[61] Bayliss R. , Sardon T., Vernos I., Conti E. Structural basis of Aurora-A activation by TPX2 at the mitotic spindle. Mol. Cell. 2003; 12:851–862. 14580337 10.1016/s1097-2765(03)00392-7

[62] Ong S.-E. , Blagoev B., Kratchmarova I., Kristensen D.B., Steen H., Pandey A., Mann M. Stable isotope labeling by amino acids in cell culture, SILAC, as a simple and accurate approach to expression proteomics. Mol. Cell Proteomics. 2002; 1:376–386. 12118079 10.1074/mcp.m200025-mcp200

[63] Smits A.H. , Jansen P.W.T.C., Poser I., Hyman A.a., Vermeulen M. Stoichiometry of chromatin-associated protein complexes revealed by label-free quantitative mass spectrometry-based proteomics. Nucleic Acids Res. 2013; 41:e28. 23066101 10.1093/nar/gks941 PMC3592467

[64] Sirbu B.M. , Couch F.B., Cortez D. Monitoring the spatiotemporal dynamics of proteins at replication forks and in assembled chromatin using isolation of proteins on nascent DNA. Nat. Protoc. 2012; 7:594–605. 22383038 10.1038/nprot.2012.010 PMC3671908

[65] Michalski A. , Damoc E., Hauschild J.P., Lange O., Wieghaus A., Makarov A., Nagaraj N., Cox J., Mann M., Horning S. Mass spectrometry-based proteomics using q exactive, a high-performance benchtop quadrupole orbitrap mass spectrometer. Mol. Cell Proteomics. 2011; 10:M111 011015. 10.1074/mcp.M111.011015 PMC3284220 21642640

[66] Kelstrup C.D. , Young C., Lavallee R., Nielsen M.L., Olsen J.V Optimized fast and sensitive acquisition methods for shotgun proteomics on a quadrupole orbitrap mass spectrometer. J. Proteome Res. 2012; 11:3487–3497. 22537090 10.1021/pr3000249

[67] Olsen J.V , Macek B., Lange O., Makarov A., Horning S., Mann M. Higher-energy C-trap dissociation for peptide modification analysis. Nat. Methods. 2007; 4:709–712. 17721543 10.1038/nmeth1060

[68] Cox J. , Mann M. MaxQuant enables high peptide identification rates, individualized p.p.b.-range mass accuracies and proteome-wide protein quantification. Nat. Biotechnol. 2008; 26:1367–1372. 19029910 10.1038/nbt.1511

[69] Cox J. , Neuhauser N., Michalski A., Scheltema R.A., Olsen J.V, Mann M. Andromeda: a peptide search engine integrated into the maxquant environment. J. Proteome. Res. 2011; 10:1794–1805. 21254760 10.1021/pr101065j

[70] Elias J.E. , Gygi S.P. Target-decoy search strategy for increased confidence in large-scale protein identifications by mass spectrometry. Nat. Methods. 2007; 4:207–214. 17327847 10.1038/nmeth1019

[71] Smyth G.K. Linear models and empirical bayes methods for assessing differential expression in microarray experiments. Stat. Appl. Genet. Mol. Biol. 2004; 3:Article3. 16646809 10.2202/1544-6115.1027

[72] Brionne A. , Juanchich A., Hennequet-Antier C. ViSEAGO: a bioconductor package for clustering biological functions using gene ontology and semantic similarity. BioData Min. 2019; 12:16. 31406507 10.1186/s13040-019-0204-1 PMC6685253

[73] Kuleshov M.V. , Jones M.R., Rouillard A.D., Fernandez N.F., Duan Q., Wang Z., Koplev S., Jenkins S.L., Jagodnik K.M., Lachmann A. et al. Enrichr: a comprehensive gene set enrichment analysis web server 2016 update. Nucleic Acids Res. 2016; 44:W90–W97. 27141961 10.1093/nar/gkw377 PMC4987924

[74] Shannon P. Cytoscape: a software environment for integrated models of biomolecular interaction networks. Genome Res. 2003; 13:2498–2504. 14597658 10.1101/gr.1239303 PMC403769

[75] Ghandi M. , Huang F.W., Jané-Valbuena J., Kryukov G.V, Lo C.C., McDonald E.R. 3rd, Barretina J., Gelfand E.T., Bielski C.M., Li H. et al. Next-generation characterization of the cancer cell line encyclopedia. Nature. 2019; 569:503–508. 31068700 10.1038/s41586-019-1186-3 PMC6697103

[76] Corsello S.M. , Nagari R.T., Spangler R.D., Rossen J., Kocak M., Bryan J.G., Humeidi R., Peck D., Wu X., Tang A.A. et al. Discovering the anticancer potential of non-oncology drugs by systematic viability profiling. Nat. Cancer. 2020; 1:235–248. 32613204 10.1038/s43018-019-0018-6 PMC7328899

[77] Gao J. , Aksoy B.A., Dogrusoz U., Dresdner G., Gross B., Sumer S.O., Sun Y., Jacobsen A., Sinha R., Larsson E. et al. Integrative analysis of complex cancer genomics and clinical profiles using the cBioPortal. Sci. Signal. 2013; 6:pl1. 23550210 10.1126/scisignal.2004088 PMC4160307

[78] Cerami E. , Gao J., Dogrusoz U., Gross B.E., Sumer S.O., Aksoy B.A., Jacobsen A., Byrne C.J., Heuer M.L., Larsson E. et al. The cBio cancer genomics portal: an open platform for exploring multidimensional cancer genomics data. Cancer Discov. 2012; 2:401–404. 22588877 10.1158/2159-8290.CD-12-0095 PMC3956037

[79] Certo M.T. , Ryu B.Y., Annis J.E., Garibov M., Jordan V., Rawlings D.J., Scharenberg A.M. Tracking genome engineering outcome at individual DNA breakpoints. Nat. Met. 2012; 8:671–676. 10.1038/nmeth.1648 PMC3415300 21743461

[80] Roukos V. , Pegoraro G., Voss T.C., Misteli T. Cell cycle staging of individual cells by fluorescence microscopy. Nat. Protoc. 2015; 10:334–348. 25633629 10.1038/nprot.2015.016 PMC6318798

[81] Bartlett E. , Bonfiglio J.J., Prokhorova E., Colby T., Zobel F., Ahel I., Matic I. Interplay of histone marks with serine ADP-Ribosylation. Cell Rep. 2018; 24:3488–3502. 30257210 10.1016/j.celrep.2018.08.092 PMC6172693

[82] Ahel D. , Hořejší Z., Wiechens N., Polo S.E., Garcia-Wilson E., Ahel I., Flynn H., Skehel M., West S.C., Jackson S.P. et al. Poly(ADP-ribose)-dependent regulation of DNA repair by the chromatin remodeling enzyme ALC1. Science. 2009; 325:1240–1243. 19661379 10.1126/science.1177321 PMC3443743

[83] Kaufmann T. , Herbert S., Hackl B., Besold J.M., Schramek C., Gotzmann J., Elsayad K., Slade D. Direct measurement of protein-protein interactions by FLIM-FRET at UV laser-induced DNA damage sites in living cells. Nucleic Acids Res. 2020; 48:E122–E122. 33053171 10.1093/nar/gkaa859 PMC7708043

[84] Sirbu B.M. , McDonald W.H., Dungrawala H., Badu-Nkansah A., Kavanaugh G.M., Chen Y., Tabb D.L., Cortez D. Identification of proteins at active, stalled, and collapsed replication forks using isolation of proteins on nascent DNA (iPOND) coupled with mass spectrometry. J. Biol. Chem. 2013; 288:31458–31467. 24047897 10.1074/jbc.M113.511337 PMC3814742

[85] Sirbu B.M. , Couch F.B., Feigerle J.T., Bhaskara S., Hiebert S.W., Cortez D. Analysis of protein dynamics at active, stalled, and collapsed replication forks. Genes Dev. 2011; 25:1320–1327. 21685366 10.1101/gad.2053211 PMC3127432

[86] Petermann E. , Orta M.L., Issaeva N., Schultz N., Helleday T. Hydroxyurea-Stalled replication forks become progressively inactivated and require two different RAD51-Mediated pathways for restart and repair. Mol. Cell. 2010; 37:492–502. 20188668 10.1016/j.molcel.2010.01.021 PMC2958316

[87] Dungrawala H. , Rose K.L., Bhat K.P., Mohni K.N., Glick G.G., Couch F.B., Cortez D. The replication checkpoint prevents two types of fork collapse without regulating replisome stability. Mol. Cell. 2015; 59:998–1010. 26365379 10.1016/j.molcel.2015.07.030 PMC4575883

[88] Gruss O.J. , Wittmann M., Yokoyama H., Pepperkok R., Kufer T., Silljé H., Karsenti E., Mattaj I.W., Vernos I. Chromosome-induced microtubule assembly mediated by TPX2 is required for spindle formation in HeLa cells. Nat. Cell Biol. 2002; 4:871–879. 12389033 10.1038/ncb870

[89] Kustatscher G. , Hégarat N., Wills K.L.H., Furlan C., Bukowski-Wills J.-C., Hochegger H., Rappsilber J. Proteomics of a fuzzy organelle: interphase chromatin. EMBO J. 2014; 33:648–664. 24534090 10.1002/embj.201387614 PMC3983682

[90] Vivelo C.A. , Wat R., Agrawal C., Tee H.Y., Leung A.K.L. ADPriboDB: the database of ADP-ribosylated proteins. Nucleic Acids Res. 2017; 45:D204–D209. 27507885 10.1093/nar/gkw706 PMC5210603

[91] Ayyappan V. , Wat R., Barber C., Vivelo C.A., Gauch K., Visanpattanasin P., Cook G., Sazeides C., Leung A.K.L. ADPriboDB v2.0: an updated database of ADP-ribosylated proteins. Nucleic Acids Res. 2021; 49:D261–D265. 33137182 10.1093/nar/gkaa941 PMC7778992

[92] Sanchez-Pulido L. , Perez L., Kuhn S., Vernos I., Andrade-Navarro M.A. The C-terminal domain of TPX2 is made of alpha-helical tandem repeats. BMC Struct Biol. 2016; 16:17. 27782824 10.1186/s12900-016-0070-8 PMC5080731

[93] Liao H. , Ji F., Helleday T., Ying S. Mechanisms for stalled replication fork stabilization: new targets for synthetic lethality strategies in cancer treatments. EMBO Rep. 2018; 19:e46263. 30108055 10.15252/embr.201846263 PMC6123652

[94] Rouleau M. , Patel A., Hendzel M.J., Kaufmann S.H., Poirier G.G. PARP inhibition: PARP1 and beyond. Nat. Rev. Cancer. 2010; 10:293–301. 20200537 10.1038/nrc2812 PMC2910902

[95] Krastev D.B. , Li S., Sun Y., Wicks A.J., Hoslett G., Weekes D., Badder L.M., Knight E.G., Marlow R., Pardo M.C. et al. The ubiquitin-dependent ATPase p97 removes cytotoxic trapped PARP1 from chromatin. Nat. Cell. Biol. 2022; 24:62–73. 35013556 10.1038/s41556-021-00807-6 PMC8760077

[96] Haahr P. , Hoffmann S., Tollenaere M.A.X., Ho T., Toledo L.I., Mann M., Bekker-Jensen S., Räschle M., Mailand N. Activation of the ATR kinase by the RPA-binding protein ETAA1. Nat. Cell. Biol. 2016; 18:1196–1207. 27723717 10.1038/ncb3422

[97] Nakamura K. , Kustatscher G., Alabert C., Hödl M., Forne I., Völker-Albert M., Satpathy S., Beyer T.E., Mailand N., Choudhary C. et al. Proteome dynamics at broken replication forks reveal a distinct ATM-directed repair response suppressing DNA double-strand break ubiquitination. Mol. Cell. 2021; 81:1084–1099. 33450211 10.1016/j.molcel.2020.12.025 PMC7939521

[98] Neumayer G. , Helfricht A., Shim S.Y., Le H.T., Lundin C., Belzil C., Chansard M., Yu Y., Lees-Miller S.P., Gruss O.J. et al. Targeting protein for xenopus kinesin-like protein 2 (TPX2) regulates γ-histone 2AX (γ-H2AX) levels upon ionizing radiation. J. Biol. Chem. 2012; 287:42206–42222. 23045526 10.1074/jbc.M112.385674 PMC3516765

[99] Ramakrishna M. , Williams L.H., Boyle S.E., Bearfoot J.L., Sridhar A., Speed T.P., Gorringe K.L., Campbell I.G. Identification of candidate growth promoting genes in ovarian cancer through integrated copy number and expression analysis. PLoS One. 2010; 5:e9983. 20386695 10.1371/journal.pone.0009983 PMC2851616

[100] Scharer C.D. , Laycock N., Osunkoya A.O., Logani S., McDonald J.F., Benigno B.B., Moreno C.S. Aurora kinase inhibitors synergize with paclitaxel to induce apoptosis in ovarian cancer cells. J. Transl. Med. 2008; 6:79. 19077237 10.1186/1479-5876-6-79 PMC2614415

[101] Vainio P. , Mpindi J.-P., Kohonen P., Fey V., Mirtti T., Alanen K.A., Perälä M., Kallioniemi O., Iljin K. High-throughput transcriptomic and RNAi analysis identifies AIM1, ERGIC1, TMED3 and TPX2 as potential drug targets in prostate cancer. PLoS One. 2012; 7:e39801. 22761906 10.1371/journal.pone.0039801 PMC3386189

[102] Hu Y. , Wu G., Rusch M., Lukes L., Buetow K.H., Zhang J., Hunter K.W. Integrated cross-species transcriptional network analysis of metastatic susceptibility. Proc. Natl. Acad. Sci. U.S.A. 2012; 109:3184–3189. 22308418 10.1073/pnas.1117872109 PMC3286991

[103] van Gijn S.E. , Wierenga E., van den Tempel N., Kok Y.P., Heijink A.M., Spierings D.C.J., Foijer F., van Vugt M.A.T.M., Fehrmann R.S.N. TPX2/Aurora kinase a signaling as a potential therapeutic target in genomically unstable cancer cells. Oncogene. 2019; 38:852–867. 30177840 10.1038/s41388-018-0470-2 PMC6367211

[104] Byrum A.K. , Carvajal-Maldonado D., Mudge M.C., Valle-Garcia D., Majid M.C., Patel R., Sowa M.E., Gygi S.P., Wade Harper J., Shi Y. et al. Mitotic regulators TPX2 and aurora a protect DNA forks during replication stress by counteracting 53BP1 function. J. Cell Biol. 2019; 218:422–432. 30602538 10.1083/jcb.201803003 PMC6363440

[105] Vizcaino J.A. , Cote R.G., Csordas A., Dianes J.A., Fabregat A., Foster J.M., Griss J., Alpi E., Birim M., Contell J. et al. The PRoteomics IDEntifications (PRIDE) database and associated tools: status in 2013. Nucleic Acids Res. 2013; 41:D1063–D1069. 23203882 10.1093/nar/gks1262 PMC3531176

