# Supplementary material for: PARP1 proximity proteomics reveals interaction partners at stressed replication forks
Source: Nucleic Acids Res. 2022 Nov 9;50(20):11600–18. doi: 10.1093/nar/gkac948 (PMC9723622; doi:10.1093/nar/gkac948)
Supplement: gkac948_Supplemental_Files [file gkac948_supplemental_files.zip › Supplementary information.pdf]

Figure S1

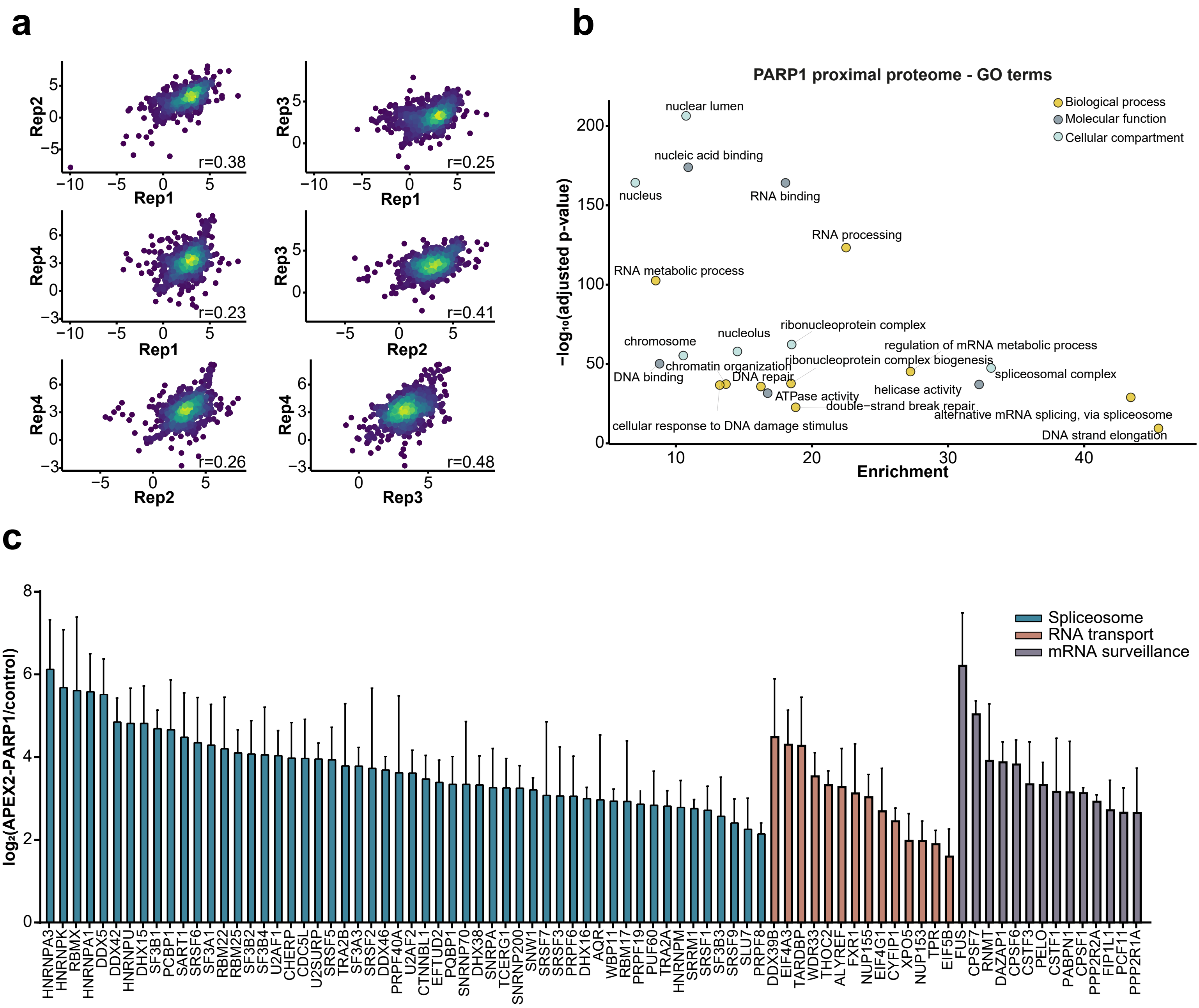

a
r=0.38
r=0.25
r=0.23
r=0.41
r=0.26
r=0.48
Rep1
Rep2
Rep3
Rep4
b
PARP1 proximal proteome - GO terms
Biological process
Molecular function
Cellular compartment
nuclear lumen
nucleic acid binding
nucleus
RNA binding
RNA processing
RNA metabolic process
ribonucleoprotein complex
chromosome
nucleolus
regulation of mRNA metabolic process
chromatin organization
ribonucleoprotein complex biogenesis
DNA binding
DNA repair
spliceosomal complex
helicase activity
ATPase activity
double−strand break repair
alternative mRNA splicing, via spliceosome
cellular response to DNA damage stimulus
DNA strand elongation
−log₁₀(adjusted p-value)
Enrichment
c
Spliceosome
RNA transport
mRNA surveillance
log₂(APEX2-PARP1/control)
HNRNPA3 HNRNPK RBMX HNRNPA1 DDX5 DDX42 HNRNPU DHX15 SF3B1 PCBP1 SART1 SRSF6 SF3A1 RBM22 RBM25 SF3B2 SF3B4 U2AF1 CHERP CDC5L U2SURP SRSF5 TRA2B SF3A3 SRSF2 DDX46 PRPF40A U2AF2 CTNNBL1 EFTUD2 PQBP1 SNRNP70 DHX38 SNRPA TCERG1 SNRNP200 SNW1 SRSF7 SRSF3 PRPF6 DHX16 AQR WBP11 RBM17 PRPF19 PUF60 TRA2A HNRNPM SRRM1 SRSF1 SF3B3 SRSF9 SLU7 PRPF8
DDX39B EIF4A3 TARDBP WDR33 THOC2 ALYREF FXR1 NUP155 EIF4G1 CYFIP1 XPO5 NUP153 TPR EIF5B
FUS CPSF7 RNMT DAZAP1 CPSF6 CSTF3 PELO CSTF1 PABPN1 CPSF1 PPP2R2A FIP1L1 PCF11 PPP2R1A

Supplementary Figure 1

a. Pearson correlations of n = 4 biologically independent experiments of PARP1 APEX2 experiments. Scatter plots display the correlation between individual replicates. Correlation is shown beside each scatter plot.
b. GO term analysis (Molecular Function, Biological Process and Cellular Compartment) of proteins proximal to PARP1with an FDR <1%. P-values were calculated by two-sided Fisher´s exact test and corrected for multiple comparisons using Benjamini-Hochberg correction.
c. Enrichment of PARP1 proximal RNA metabolism proteins contributing to the KEGG pathways in Figure 1c. Data are represented as mean +/- standard deviation. Colors indicate the depicted KEGG pathways.

Figure S2

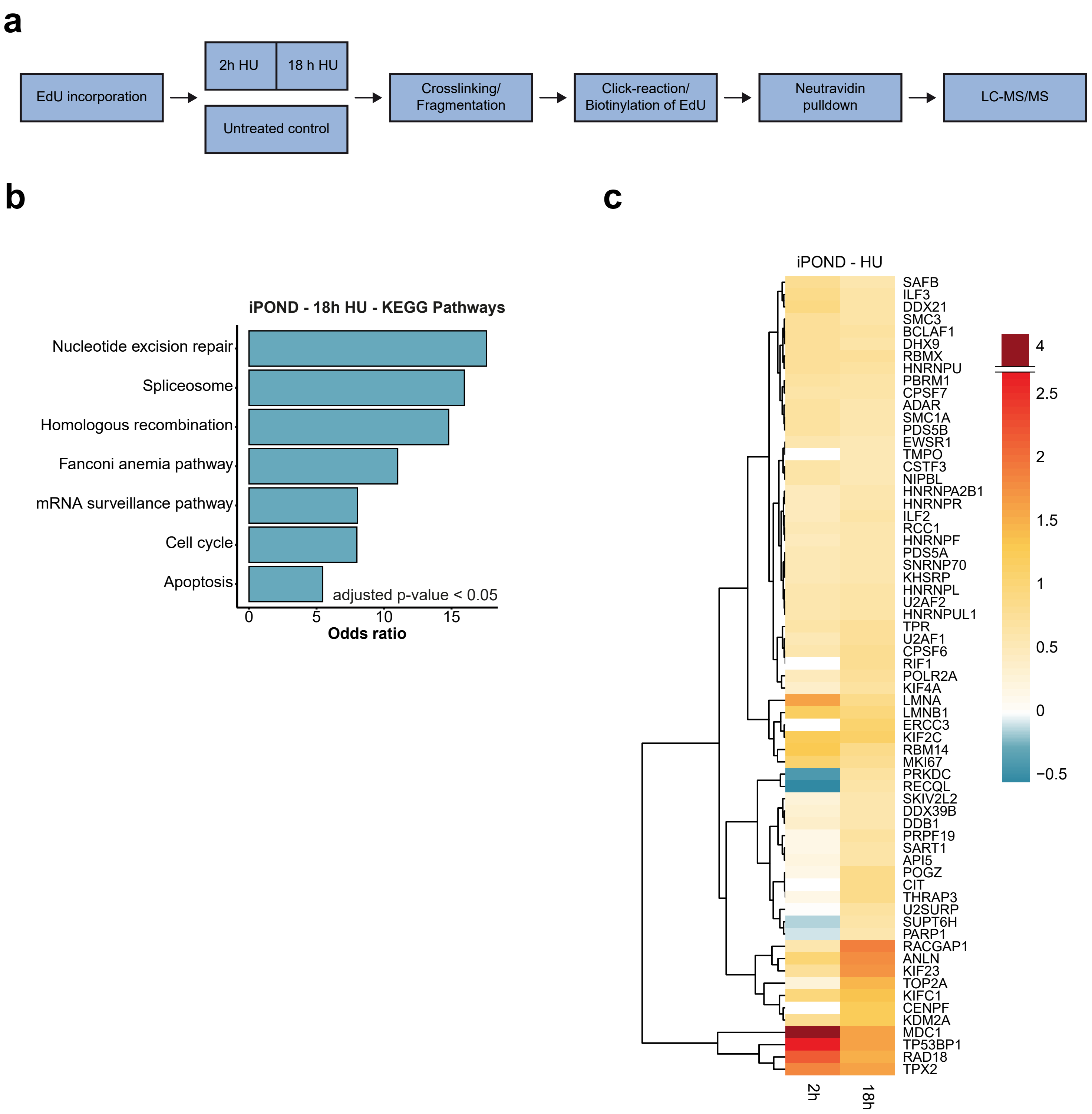
a
EdU incorporation
2h HU
18 h HU
Untreated control
Crosslinking/ Fragmentation
Click-reaction/ Biotinylation of EdU
Neutravidin pulldown
LC-MS/MS
b
iPOND - 18h HU - KEGG Pathways
Nucleotide excision repair
Spliceosome
Homologous recombination
Fanconi anemia pathway
mRNA surveillance pathway
Cell cycle
Apoptosis
adjusted p-value < 0.05
0
5
10
15
Odds ratio
c
iPOND - HU
SAFB
ILF3
DDX21
SMC3
BCLAF1
DHX9
RBMX
HNRNPU
PBRM1
CPSF7
ADAR
SMC1A
PDS5B
EWSR1
TMPO
CSTF3
NIPBL
HNRNPA2B1
HNRNPR
ILF2
RCC1
HNRNPF
PDS5A
SNRNP70
KHSRP
HNRNPL
U2AF2
HNRNPUL1
TPR
U2AF1
CPSF6
RIF1
POLR2A
KIF4A
LMNA
LMNB1
ERCC3
KIF2C
RBM14
MKI67
PRKDC
RECQL
SKIV2L2
DDX39B
DDB1
PRPF19
SART1
API5
POGZ
CIT
THRAP3
U2SURP
SUPT6H
PARP1
RACGAP1
ANLN
KIF23
TOP2A
KIFC1
CENPF
KDM2A
MDC1
TP53BP1
RAD18
TPX2
2h
18h
4
2.5
2
1.5
1
0.5
0
−0.5

Supplementary Figure 2

a. Workflow of iPOND experiment comparing replication fork associated proteins of unchallenged cells or after treatment with 2 mM HU (2h, 18h). Nascent DNA was labelled by treating cells with 10 µM EdU for 30 min. After formaldehyde fixation and subsequent biotin conjugation cells were subjected to sonication. Biotinylated chromatin fragments were purified using neutravidin and replication fork associated proteins de-crosslinked and afterwards analyzed by LC-MS/MS.

b. Bar plot representing the top 10 enriched KEGG pathways of proteins enriched at persistently stalled replication forks (18 h HU) with an FDR <5%. Pathways are ranked based on their odds ratio from EnrichR. All represented terms have an adjusted p-value below 0.01 calculated using Fisher´s exact test with Benjamini-Hochberg correction.

c. Heatmap showing the recruitment of the 65 proteins present in PARP1 proximal proteome and at stressed replication forks (2 h and 18 h HU). Log_2 ratios between HU/untreated are plotted color coded from blue to red.

Figure S3

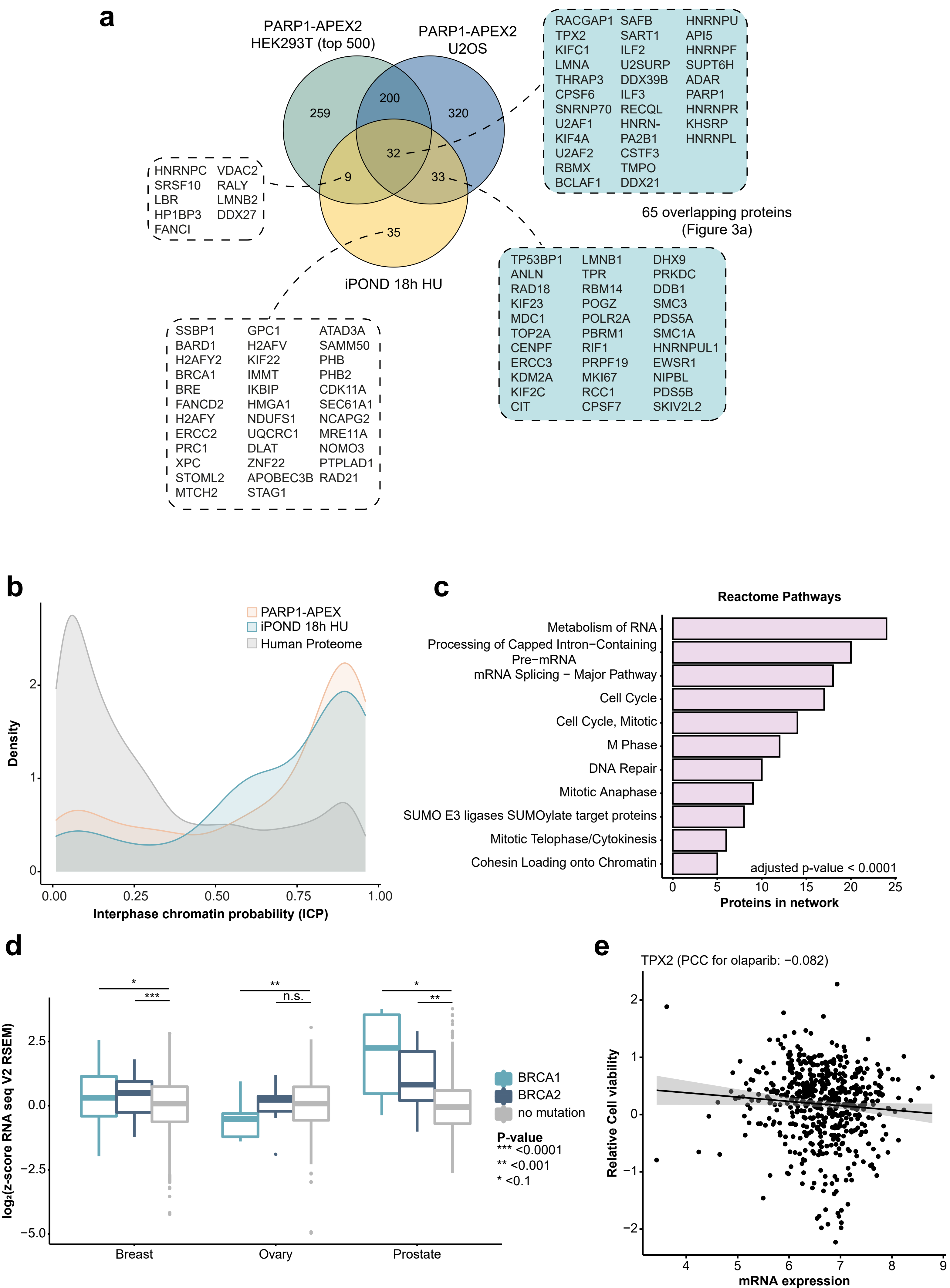
a
PARP1-APEX2 HEK293T (top 500)
PARP1-APEX2 U2OS
iPOND 18h HU
259
200
320
32
9
33
35
RACGAP1 SAFB HNRNPU
TPX2 SART1 API5
KIFC1 ILF2 HNRNPF
LMNA U2SURP SUPT6H
THRAP3 DDX39B ADAR
CPSF6 ILF3 PARP1
SNRNP70 RECQL HNRNPR
U2AF1 HNRN-PA2B1 KHSRP
KIF4A HNRNPL
U2AF2 CSTF3
RBMX TMPO
BCLAF1 DDX21
65 overlapping proteins (Figure 3a)
HNRNPC VDAC2
SRSF10 RALY
LBR LMNB2
HP1BP3 DDX27
FANCI
TP53BP1 LMNB1 DHX9
ANLN TPR PRKDC
RAD18 RBM14 DDB1
KIF23 POGZ SMC3
MDC1 POLR2A PDS5A
TOP2A PBRM1 SMC1A
CENPF RIF1 HNRNPUL1
ERCC3 PRPF19 EWSR1
KDM2A MKI67 NIPBL
KIF2C RCC1 PDS5B
CIT CPSF7 SKIV2L2
SSBP1 GPC1 ATAD3A
BARD1 H2AFV SAMM50
H2AFY2 KIF22 PHB
BRCA1 IMMT PHB2
BRE IKBIP CDK11A
FANCD2 HMGA1 SEC61A1
H2AFY NDUFS1 NCAPG2
ERCC2 UQCRC1 MRE11A
PRC1 DLAT NOMO3
XPC ZNF22 PTPLAD1
STOML2 APOBEC3B RAD21
MTCH2 STAG1
b
PARP1-APEX
iPOND 18h HU
Human Proteome
Density
Interphase chromatin probability (ICP)
0.00 0.25 0.50 0.75 1.00
c
Reactome Pathways
Metabolism of RNA
Processing of Capped Intron−Containing Pre−mRNA
mRNA Splicing − Major Pathway
Cell Cycle
Cell Cycle, Mitotic
M Phase
DNA Repair
Mitotic Anaphase
SUMO E3 ligases SUMOylate target proteins
Mitotic Telophase/Cytokinesis
Cohesin Loading onto Chromatin
adjusted p-value < 0.0001
Proteins in network
0 5 10 15 20 25
d
log₂(z-score RNA seq V2 RSEM)
Breast
Ovary
Prostate
n.s.
BRCA1
BRCA2
no mutation
P-value
*** <0.0001
** <0.001
* <0.1
e
TPX2 (PCC for olaparib: −0.082)
Relative Cell viability
mRNA expression

Supplementary Figure 3

a. Venn diagram displaying the overlap between PARP1-APEX2 in U2OS and HEK293T cells and iPOND after 18 h HU treatment in U2OS cells.
b. Density plots displaying the interphase chromatin probability (IPC) of proteins identified by PARP1 APEX (orange) and 18 h HU iPOND (blue). All proteins with assigned IPC are plotted in grey as control.
c. Reactome pathway enrichment analysis of 65 proteins proximal to PARP1 and present at stressed replication forks. Bar plot representing the terms with an adjusted p-value < 0.0001 calculated with two-sided Fisher´s exact test with Bonferroni correction. Significant pathways are sorted based on the number of proteins in the network that contribute to the respective Reactome pathway.
d. Log_2 of z-scored RSEM TPX2 mRNA expression levels in *BRCA1/2* positive and negative breast, ovarian and prostate cancers extracted from the cBioPortal. Center of boxplots indicate the median, limits the 25th-75th percentile and whiskers the 10th-90th percentile. P-values (*p < 0.1, **p < 0.001, ***p < 0.0001) were derived using T-test and corrected for multiple comparisons using Benjamini-Hochberg correction.
e. Scatter plot displaying Pearson correlation (black line) between TPX2 mRNA expression and sensitivity to olaparib of 588 cancer cell lines derived from the DepMap portal. Black dots indicate each individual cell line. Pearson correlation coefficient (PCC) = -0.082.

Figure S4

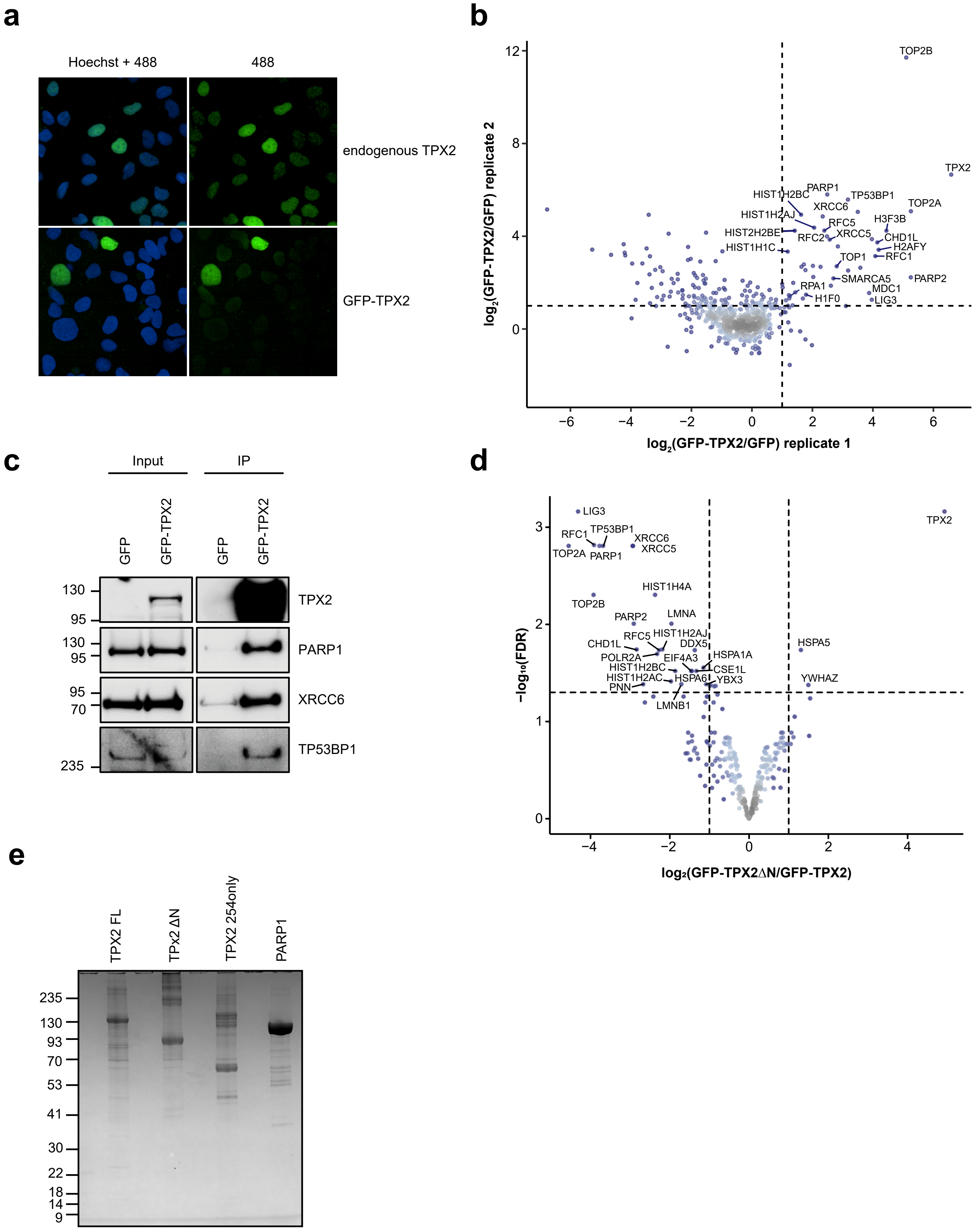

a
Hoechst + 488
488
endogenous TPX2
GFP-TPX2
b
log2(GFP-TPX2/GFP) replicate 2
log2(GFP-TPX2/GFP) replicate 1
TOP2B
TPX2
PARP1
HIST1H2BC
TP53BP1
XRCC6
TOP2A
HIST1H2AJ
RFC5
H3F3B
HIST2H2BE
RFC2
XRCC5
CHD1L
H2AFY
HIST1H1C
TOP1
RFC1
SMARCA5
PARP2
RPA1
MDC1
H1F0
LIG3
c
Input
IP
GFP
GFP-TPX2
TPX2
PARP1
XRCC6
TP53BP1
130
95
70
235
d
−log10(FDR)
log2(GFP-TPX2ΔN/GFP-TPX2)
LIG3
TPX2
RFC1
TP53BP1
XRCC6
XRCC5
TOP2A
PARP1
HIST1H4A
TOP2B
LMNA
PARP2
HIST1H2AJ
RFC5
DDX5
HSPA5
CHD1L
POLR2A
EIF4A3
HSPA1A
HIST1H2BC
CSE1L
HIST1H2AC
HSPA6
YBX3
YWHAZ
PNN
LMNB1
e
TPX2 FL
TPx2 ΔN
TPX2 254only
PARP1
235
130
93
70
53
41
30
22
18
14
9

Supplementary Figure 4

a. Representative immunofluorescence images of endogenous TPX2 compared to exogenously expressed GFP-tagged TPX2. DNA was counterstained with Hoechst33342 (blue).
b. SILAC-based co-IP-MS using GFP-tagged TPX2 as bait with a label switch against GFP control in n = 2 biologically independent experiments. Selected proteins with a fold change > 2 are highlighted. Dotted lines indicate fold change >2.
c. Western blot analysis of transiently expressed GFP and GFP-tagged TPX2 that were immunoprecipitated with a GFP-Trap. Interacting proteins were detected in the input and IP using indicated antibodies.
d. SILAC-based co-IP-MS analysis comparing interactors of full-length TPX2 and ΔN mutant. Both TPX2 variants were transiently expressed in U2OS cells before GFP-IP from whole cell lysates. Proteins of interest with a fold change >2 and FDR< 5% determined by limma are highlighted. Dotted lines indicate FC >2 and FDR <5%.
e. Coomassie staining of purified PARP1 and His-GFP-tagged TPX2 variants (full length, ΔN and 1-254 only).

Figure S5

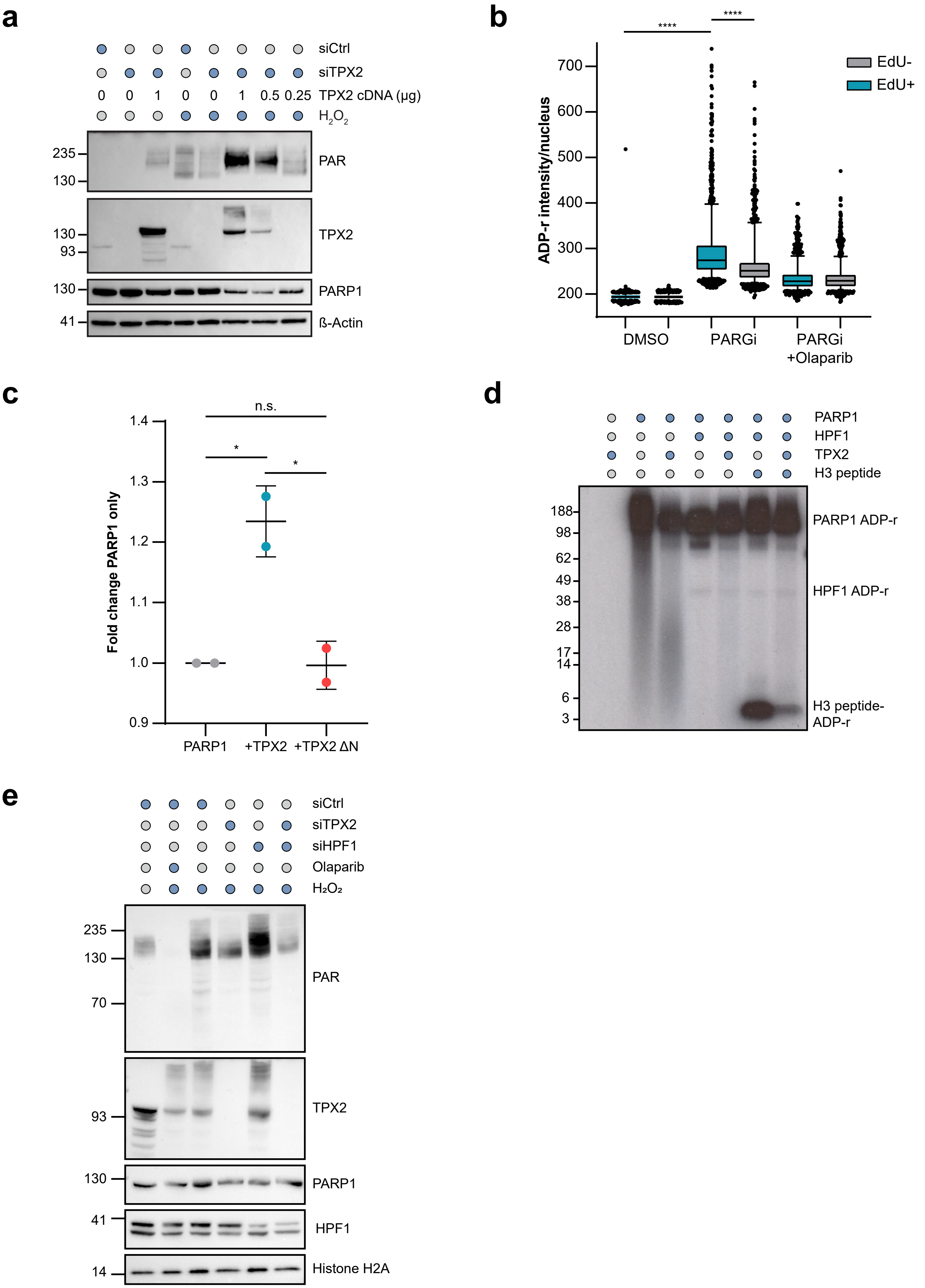
a
siCtrl
siTPX2
TPX2 cDNA (µg)
0 0 1 0 0 1 0.5 0.25
H_2O_2
235
130
PAR
130
93
TPX2
130
PARP1
41
ß-Actin
b
****
****
ADP-r intensity/nucleus
700
600
500
400
300
200
EdU-
EdU+
DMSO
PARGi
PARGi
+Olaparib
c
n.s.
*
*
Fold change PARP1 only
1.4
1.3
1.2
1.1
1.0
0.9
PARP1
+TPX2
+TPX2 ΔN
d
PARP1
HPF1
TPX2
H3 peptide
188
98
62
49
38
28
17
14
6
3
PARP1 ADP-r
HPF1 ADP-r
H3 peptide-
ADP-r
e
siCtrl
siTPX2
siHPF1
Olaparib
H_2O_2
235
130
70
PAR
93
TPX2
130
PARP1
41
HPF1
14
Histone H2A

Supplementary Figure 5

a. Western blot displaying the effect of different amounts (0-1 µg cDNA) of exogenously expressed, siRNA-resistant GFP-tagged TPX2 on H_2O_2-induced PARylation. U2OS cells were treated with 2 mM H_2O_2 for 10 minutes after 48h control or TPX2 siRNA knockdown.

b. Representative box plot of n = 2 biologically independent experiments displaying the mean ADP-ribosylation per nucleus in EdU negative (grey) and EdU positive (turquoise) cells. Cells were treated with 10 mM PARGi for 1 h +/- addition of 1 µM olaparib. The center of the boxplots indicate the median, limits the 25th-75th percentile, whiskers the 10th-90th percentile and dots indicate outliers. ****P-value < 0.0001, One-way ANOVA with Tukey correction for multiple comparisons.

c. Dot blot displaying the mean +/- SD of n = 2 biologically independent experiments of the *in vitro* ADP-ribosylation Western blot analysis with purified PARP1 and GFP-TPX2 proteins (full-length and ΔN mutant). Individual measurements are represented as dots.

d. PARP1 activity *in vitro* ADP-ribosylation assay in the presence or absence of TPX2, HPF1, or an H3 peptide. The higher molecular weight signal corresponds to PARylated PARP1 and a lower molecular signal to H3 peptide PARylation. ADP-ribosylation is triggered by the addition of radioactive phosphorus-containing (P_{32}) NAD^+. Blue spots on top indicate the presence of a protein in the respective condition.

e. Western blot analysis of ADP-ribosylation in U2OS cells that were either transfected with a control siRNA, siRNA targeting TPX2, or HPF1. Oxidative stress was induced by 2 mM H_2O_2 treatment for 10 minutes.

Figure S6

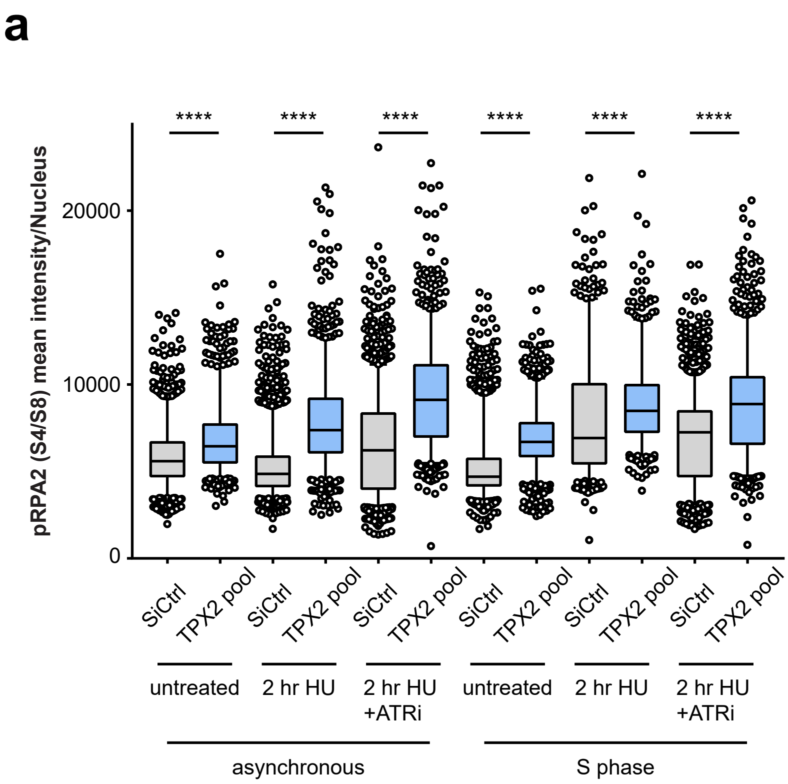
a
pRPA2 (S4/S8) mean intensity/Nucleus
20000
10000
0
****
SiCtrl
TPX2 pool
untreated
2 hr HU
2 hr HU +ATRi
asynchronous
S phase

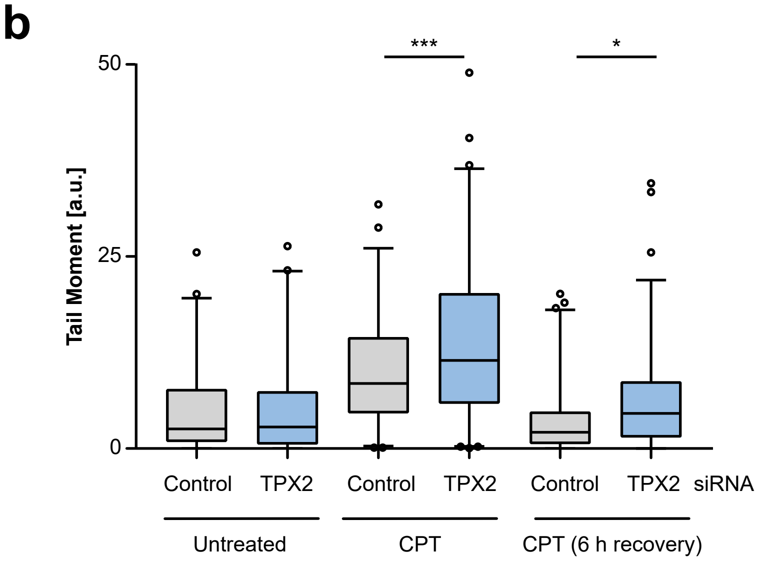
b
Tail Moment [a.u.]
50
25
0
***
*
Control
TPX2
siRNA
Untreated
CPT
CPT (6 h recovery)

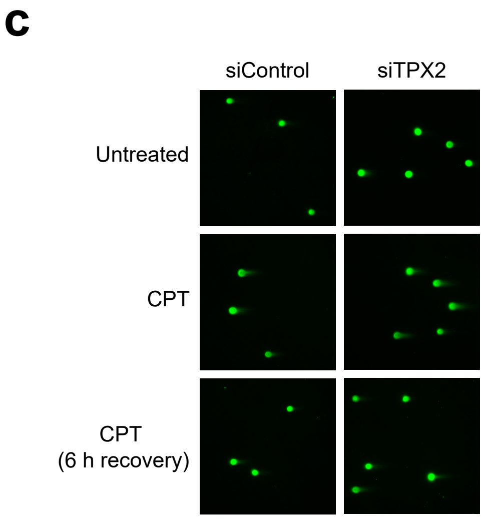
c
siControl
siTPX2
Untreated
CPT
CPT
(6 h recovery)

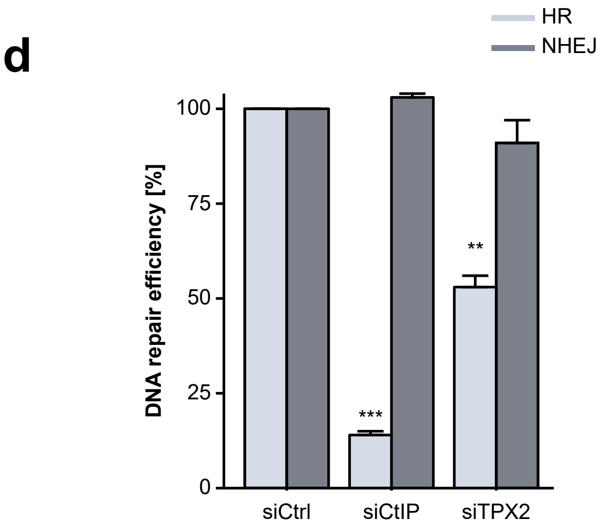
d
HR
NHEJ
DNA repair efficiency [%]
100
75
50
25
0
***
**
siCtrl
siCtIP
siTPX2

Supplementary Figure 6

a. Quantification of the mean pRPA intensity per nucleus based on immunofluorescence analysis. Experimental setup as in Fig. 6c. Center of boxplots indicate the median, limits the 25th-75th percentile, whiskers the 10th-90th percentile and dots indicate outliers. ****P-value < 0.0001, One-way ANOVA with Tukey correction for multiple comparisons.
b. Neutral comet assay 48 h after indicated knockdowns. U2OS cells were treated for 2 h (6 h recovery) with 10 µM CPT before being immobilized in low melting agarose. Boxplot displaying the tail moment quantification of n > 50 cells. Center of boxplots indicate the median, limits the 25th-75th percentile, whiskers the 10th-90th percentile and dots indicate outliers. P-values (* < 0.05, *** < 0.001) were derived using one-way-ANOVA with Tukey correction for multiple comparisons.
c. Example images of comets after control or TPX2 knockdown and indicated CPT treatments corresponding to Figure S6a.
d. Flow cytometry-based Traffic Light Reporter (TLR) assay in U2OS cells after 72 h of indicated siRNA knockdowns. Cells were transfected with a GFP donor plasmid and I-SceI expression vector 6 h after siRNA transfection. Homologous recombination (HR) and non-homologous end joining (NHEJ) repair efficiency from n = 3 biologically independent experiments are displayed normalized to the control knockdown. Data are represented as mean +/- standard deviation. **p-value < 0.01, ***p-value < 0.001, one-way-ANOVA with Tukey correction for multiple comparisons.

Supplementary Tables

Supplementary Table 1

The excel sheet contains a spreadsheet with all quantified protein groups in PARP1 nuclear proximal proteome from U2OS cells with an FDR <1%.

Supplementary Table 2

The excel sheet contains a spreadsheet with all quantified protein groups in PARP1 nuclear proximal proteome from HEK293T cells. It includes two distinct experimental setups within the same triple SILAC analysis: (+H_2O_2/-H_2O_2) and (DMSO+H_2O_2/olaparib + H_2O_2).

Supplementary Table 3

The excel sheet contains a spreadsheet with all quantified protein groups in 18 h HU iPOND experiment from U2OS cells with an FDR <5%.

Supplementary Table 4

The excel sheet contains a spreadsheet with all quantified protein groups from LFQ GFP-TPX2 (bait) vs GFP (control) MS co-IP experiment from U2OS cells with indicated significances and fold changes.
